# *Plasmodium falciparum* sexual differentiation in malaria patients is associated with host factors and GDV1-dependent genes

**DOI:** 10.1038/s41467-019-10172-6

**Published:** 2019-05-13

**Authors:** Miho Usui, Surendra K. Prajapati, Ruth Ayanful-Torgby, Festus K. Acquah, Elizabeth Cudjoe, Courage Kakaney, Jones A. Amponsah, Evans K. Obboh, Deepti K. Reddy, Michelle C. Barbeau, Lacy M. Simons, Beata Czesny, Sorana Raiciulescu, Cara Olsen, Benjamin K. Abuaku, Linda E. Amoah, Kim C. Williamson

**Affiliations:** 1 Uniformed Services University of the Health Sciences, Bethesda, MD 20814 USA; 2 Loyola University Chicago, Chicago, IL 60660 USA; 3 Noguchi Memorial Institute for Medical Research, University of Ghana, Accra, Ghana; 4 School of Medical Sciences, University of Cape Coast, Cape Coast, Ghana; 5 Present Address: Uniformed Services University of the Health Sciences, Department of Microbiology and Immunology, 4301 Jones Bridge Rd, Bethesda, MD 20814 USA

**Keywords:** Differentiation, Infectious-disease epidemiology, Infectious-disease diagnostics, Malaria

## Abstract

*Plasmodium* sexual differentiation is required for malaria transmission, yet much remains unknown about its regulation. Here, we quantify early gametocyte-committed ring (gc-ring) stage, *P. falciparum* parasites in 260 uncomplicated malaria patient blood samples 10 days before maturation to transmissible stage V gametocytes using a gametocyte conversion assay (GCA). Seventy six percent of the samples have gc-rings, but the ratio of gametocyte to asexual-committed rings (GCR) varies widely (0–78%). GCR correlates positively with parasitemia and is negatively influenced by fever, not hematocrit, age or leukocyte counts. Higher expression levels of GDV1-dependent genes, *ap2-g*, *msrp1* and *gexp5*, as well as a *gdv1* allele encoding H^217^ are associated with high GCR, while high plasma lysophosphatidylcholine levels are associated with low GCR in the second study year. The results provide a view of sexual differentiation in the field and suggest key regulatory roles for clinical factors and *gdv1* in gametocytogenesis in vivo.

## Abstract (web summary)

Here, the authors quantify early gametocyte-committed ring (gc-ring) stage *Plasmodium falciparum* parasites in 260 malaria patients 10 days before maturation to transmissible stage V gametocytes, and show that the ratio of circulating gc-rings is positively correlated with parasitemia and negatively correlated with body temperature.

## Introduction

Controlling malaria transmission is key to global malaria eradication efforts and continues to be highlighted as a critical research area by the recent Malaria Eradication (MalERA) refresh panel^1^. Human to human malaria transmission via a mosquito requires the production of sexual stage parasites, called gametocytes, which are produced during the erythrocytic cycle. After erythrocyte invasion by a merozoite^2^, the parasite replicates producing new merozoites that will either continue asexual multiplication or develop into gametocytes during the next erythrocytic cycle^3–6^. The regulation of this balance between asexual propagation and sexual differentiation is key to understanding the dynamics of malaria transmission and pathology in human hosts^7,8^. After a *P. falciparum* gametocyte-committed merozoite invades an erythrocyte, it differentiates over ~10–12 days from a ring stage parasite through 5 distinct morphologic stages (I–V) into a single mature male or female gametocyte^9^. For malaria transmission a male and a female gametocyte must be taken up in a blood meal by a mosquito, fertilize and begin differentiation into sporozoites that can be spread to another human host^10^.

In the human host, immature stage I–IV *P. falciparum* gametocytes sequester and cannot be detected in peripheral blood samples, making it difficult to monitor sexual differentiation in the field until mature, transmissible stage V gametocytes are released into the circulation 8–10 days later^11–14^. This delay in the ability to monitor early gametocyte formation makes it challenging to define the factors that influence gametocytogenesis in patients before they become infectious^15,16^. Studies of stage V gametocyte prevalence in the field have clearly demonstrated wide variability between individuals^12^, but it is hard to know if this is due to gametocyte production or survival. An association between stage V gametocyte levels and low hematocrit^17,18^ or a history of illness >2 days^15,19^ has been reported, but whether these factors are causal is difficult to determine^20,21^.

In culture, both asexual and sexual differentiation are intrinsic parts of the life cycle with each successive erythrocytic cycle producing a mixture of schizonts with merozoites destined for either gametocyte production or asexual replication^22–24^ suggesting an underlying stochastic or constitutive control mechanism. However, differences in sexual differentiation rates between *P. falciparum* strains can range from 0 to 20%^25,26^, suggesting parasite genetics play a role and variation within strains also indicates modulation by host factors such as lysophosphatidylcholine (LysoPC) levels^5,21,27^. Again, the underlying regulatory mechanisms remain unknown. Two genes have been reported to be required for *P. falciparum* gametocyte production in vitro, *gametocyte development protein 1* (*gdv1*, PF3D7_0935400)^24^ and the *apetela*-2 transcription factor (*ap2-g*, PF3D7_1222600)^25,28^. The activity of *gdv1* was originally demonstrated by genetic complementation of a gametocyte-deficient line (G_def_) that had a spontaneous deletion in the *gdv1* locus on chromosome 9^24^. *Gdv1* deletions are frequently associated with the loss of gametocyte production in lab strains, while overexpression increases gametocyte production^24^. Field populations also exhibit high *gdv1* allelic divergence that has been suggested to vary with transmission patterns^29^. Transcription factor *ap2-g* was also identified as the target of a mutation in a gametocyte-deficient parasite line in both *P. falciparum*^28^ and *P. berghei*^30^. Notably, AP2-G protein has been shown to enhance its own transcription^25,28,30^ as well as to be epigenetically repressed by heterochromatin protein 1 (HP1, PF3D7_1220900) in schizonts producing asexually committed merozoites^31,32^. Recently, episomal expression of GDV1-tagged with green fluorescent protein (GFP) and a ligand stabilized-degradation domain (DD) in a parasite line with an intact *gdv1* locus has been shown to increase gametocyte production^33^. The GFP.DD-tagged GDV1 also associated with HP1 and localized to heterochromatin sites throughout the genome, including the *ap2-g* locus suggesting that GDV1 and HP1 interact to regulate gametocyte production.

In the work reported here, we developed a gametocyte conversion assay (GCA) to quantify gametocyte-committed ring stage parasites in human blood samples, which allows direct comparison of sexual differentiation rates with clinical characteristics and gene expression levels. Gametocytes were detected in 76% of the samples from uncomplicated malaria patients and there was a wide range of asexual- to gametocyte-committed ring ratios (GCR). GCR was positively associated with parasitemia and negatively influenced by temperature. RNA levels of *ap2-g*, AP2-G-dependent gene,* msrp1* (PF3D7_1335000)^24,25^ and AP2-G-independent gametocyte-specific gene, *gexp5* (PF3D7_0936600)^34,35^ were also higher in high GCR samples. To more carefully analyze the regulation of gametocytogenesis, we generated a parasite line that allowed tight control of gametocyte production by tagging the endogenous *gdv1* gene with GFP and a ligand-dependent degradation domain (*Pfgdv1.gfp.dd)*. In the absence of Shield 1 ligand (Shld1), *Pfgdv1.gfp.dd* gametocyte production was markedly decreased. Using this clonal *Pfgdv1.gfp.dd* line to perform GDV1 protein dosing experiments, we demonstrate that GDV1 acts as a ‘molecular rheostat’ during schizogony to control gametocyte production and gene expression. Together the data suggest that in humans there is a low basal level of GCR that is modulated by an interplay of parasitemia, body temperature, and *P. falciparum gdv1*.

## Results

### Gametocyte-committed ring quantification in patient samples

**Fig. 1:**
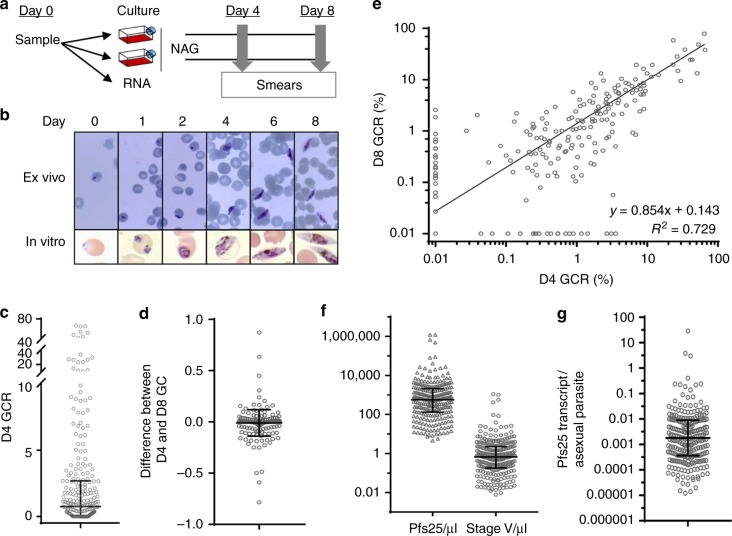
Gametocyte-committed ring quantification in patient samples. **a** Schematic of the GCA experimental set up. Each blood sample from an uncomplicated malaria patient was divided into 3 aliquots, 1 for a blood smear and clinical analysis, 1 for RNA isolation and the rest used for ex vivo culture in the presence of NAG. Culture smears were made daily to monitor parasite development. Parasitemia was quantified on Days 0, 4 and 8 and the GCR was calculated by dividing the stage II/III gametocytemia of D4 or the stage III/IV/V gametocytemia on D8 by the D0 ring stage parasitemia. **b** Giemsa-stained smears of a representative ex vivo sample and in vitro culture of the lab-adapted strain NF54. **c** The D4 GCR for each sample and the median and interquartile range is shown. **d** The difference between the D4 and D8 gametocytemias (GC) is plotted for each sample and the median and interquartile range is indicated. **e** Each subject’s D4 and D8 GCR data is plotted on the X and Y axis, respectively, and the trend line and correlation coefficient (*R*^2^) are indicated (*n* = 237). **f** *Pfs25* RNA levels were determined for each D0 blood sample using RT-qPCR and the number of *Pfs25* transcripts and stage V gametocytes per μl of blood (*Pfs25/*μl and Stage V/μl, respectively) were calculated based on standard curves using plasmid containing a single copy of *Pfs25* and purified stage V gametocytes. The *Pfs25/*μl and Stage V/μl values for each sample are plotted and the median and interquartile range are indicated. The four samples with undetectable *Pfs25* RNA levels are not included on the graph. **g** The *Pfs25* transcript levels per asexual parasite present on D0 was calculated for each sample by dividing the number of *Pfs25* transcripts/μl by the number of D0 ring stage parasites/μl determined by Giemsa-stained thick smear. The data from each sample that contained detectable *Pfs25* transcript and the median and interquartile range is shown

To directly assess gc-rings in patient blood samples, we developed an ex vivo GCA to screen blood from uncomplicated malaria patients (0.5–13 years old) presenting at Ewim Health Center in Cape Coast, Ghana during two malaria seasons, July–August 2016 and 2017 (Fig. 1 and Supplementary Table 1). Briefly, one aliquot of a subject’s blood sample is used to quantify white blood cells (WBC) and hematocrit, another is used for RNA and DNA isolation, while the remaining material is cultured ex vivo in the presence of n-acetyl glucosamine (NAG) to block further asexual replication (Fig. 1a). The cultures are followed by Giemsa-stained smears over the next 8 days for gametocyte development. Only 1 sample in 2016 and none in 2017 had microscopically detectable stage V gametocytes on a thick blood smear made on Day (D) 0 and by D4 stage V gametocytes were not detected in the ex vivo smear. Schizonts are also not observed in the NAG-treated ex vivo cultures indicating NAG had successfully blocked asexual development. Morphologically distinct stage II gametocytes are occasionally observed on D3 of ex vivo culture and became the predominant stage by D4. The parasites continue to progress to a mixture of III–V on D8, which is similar to the in vitro time course of standard lab-adapted cultures (Fig. 1b). Most of the gametocytes mature from stage II to III–V (Fig. 1c) with a median difference between the D4 stage II–III and the D8 stage III–V gametocytemia of 0 (interquartile range −0.02 to 0.008, Fig. 1d), which is comparable to that seen in standard in vitro culture. GCR is calculated by dividing the D4 stage II and III gametocytemia or the D8 stage III–V gametocytemia by the total ring stage parasitemia on Day 0 (D0P).

As an internal check of the GCR calculations we compared the D4 and D8 gametocyte conversion rates for each sample and found they are significantly positively correlated both by Pearson and regression analysis (*R*^2^ = 0.73, *p* < 0.00001) providing confidence in the counting method used to identify gametocytes (Fig. 1e). The GCR distribution for both collection years is also similar with a total of 74% and 79% of the ex vivo samples in 2016 and 2017, respectively, containing detectable gametocytes. While 20% of the samples both years have GCR above 4%, the median GCR is only 0.6% in 2016 and 0.86% in 2017 consistent with high gametocyte formation in only a small number of individuals (Fig. 1c).

The D0 blood samples are also assessed for the presence of mature gametocytes using RT-qPCR to determine *Pfs25* (PF3D7_1031000 [https://plasmodb.org/plasmo/app/record/gene/PF3D7_1031000]) transcript levels. As with the GCR a majority of the population have low levels of circulating stage V gametocytes and there is a large range of *Pfs25* transcript levels from undetectable to 1,205,521/μl blood which corresponds to 1102 stage V/μl (Fig. 1f and g and Supplementary Fig. 1). In total 26% of the samples have greater than 2 gametocytes per µl, while only 4 samples have > 100 gametocytes per μl and the median is 0.638 stage V gametocytes per μl with an interquartile range of 0.18–2.20. The sample with the highest *Pfs25* RNA level is also the one with microscopically detectable gametocytes. However, high stage V gametocyte levels on enrollment in the study (D0) do not predict high GCR demonstrating the need to further evaluate this association longitudinally in asymptomatic patients that do not have to be treated.

### Host/parasite factors modulating in vivo gametocytogenesis

**Fig. 2:**
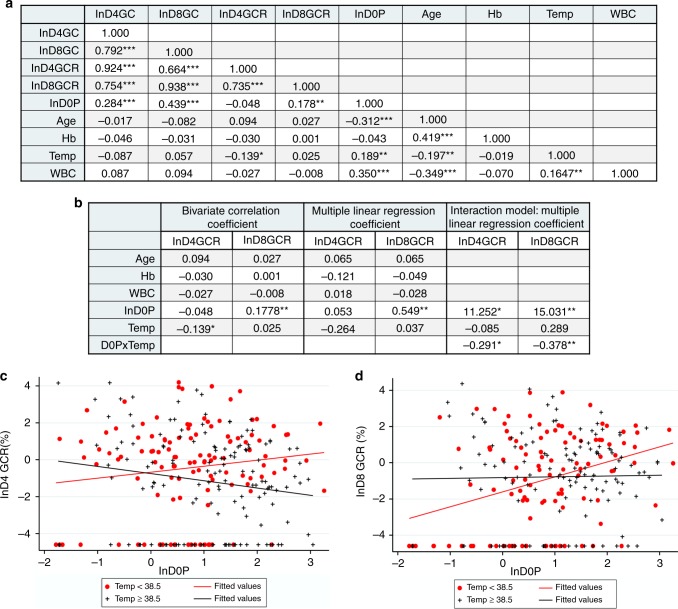
Final model of the influence of D0 parasitemia and fever on gametocyte conversion. **a** Pearson correlation of the subject’s gametocyte production, D4 and D8 gametocytemia (GC), GCR, and clinical characteristics, D0 parasitemia (D0P), hemoglobin concentration (Hb), temperature (Temp) and white blood cell count (WBC). The D0P and D4 and D8 GC and GCR were normalized by natural log transformation (ln) prior to statistical analysis. The Pearson correlation coefficient is shown and probability is indicated by *p* ≤ 0.05 (*), *p* ≤ 0.01 (**), *p* ≤ 0.001 (***). **b** Summary of the statistical tests used to evaluate the association of D4 and D8 GCR with clinical parameters. The complete table for the bivariate correlation is shown in **a**, the multiple linear regression is in Supplementary Table 2 and the interaction model is in Supplementary Table 3. The coefficients are shown with asterisks indicating p values as described for **a**. **c**, **d** Graphical representation of the negative influence of temperature on the positive relationship between the D0P and GCR. The natural log of the D4 (*n* = 260) or D8 (*n* = 238) GCR are plotted against the natural log of the D0 parasitemia (D0P). Data and trend lines from subjects with temperatures < 38.5 °C (the median D0 temperature) are shown in red dots and ≥ 38.5 °C are shown in black crosses

The relationship between patient age, temperature, parasitemia, WBCs levels, and hematocrit on D0 and gametocyte production and conversion on D4 or D8 is evaluated first by Pearson correlation (Fig. 2a) to identify significant bivariate associations. Before analysis gametocytemia, GCR and D0P are log transformed to normalize the distribution as assessed by histogram and normal quantile plots^36^. As previously reported^37^, D0 parasitemia is positively correlated with temperature and WBCs levels and negatively correlated with age. Age is also negatively correlated with temperature and WBCs and positively correlated with hematocrit. This pattern is consistent with the age dependent development of partial immunity that reduces parasite growth resulting in lower D0P and a decrease in associated symptoms, including elevated temperature and WBC counts and decreased hematocrit. An age dependent change in the pyrogenic threshold has previously been reported for malaria^38,39^ and could contribute to the negative correlation between age and temperature. Although stage V gametocyte carriage has been negatively associated with hematocrit and age^15,40^, we find no significant correlation between D4 or D8 gametocytemia or GCR and hematocrit, age, or WBC levels. D0P is positively correlated with gametocytemia on D4 and D8 (*p* < 0.001, D4GC *R*^2^ = 0.284 & D8GC *R*^2^ = 0.439), as well as D8GCR (*p* = 0.006, *R*^2^ = 0.178) by Pearson correlation, while D4 conversion rates are weakly negatively correlated with temperature (*R*^2^ = −0.139, *p* = 0.028) (Fig. 2a). To determine whether the significant associations with D4 and D8 GCR persist after adjusting for other variables, we performed a multiple linear regression analysis (Supplementary Table 2). D0P remains positively associated with D8 GCR as well as D4 and D8 gametocytemia (D8GCR *p* = 0.002 and D4GC & D8GC *p* < 0.001) and there is still a negative association between temperature and D4 GCR (*R*^2^ = −0.264, *p* = 0.069), but it did not reach *p* < 0.050 suggesting the contribution of the other factors. To further investigate the relationship between GCR and D0P and D0 temperature, we tested for an interaction between natural log normalized D0P and D0 temperatures by regression analysis using a continuous temperature model (Supplementary Table 3). The results indicate a significant negative interaction between D0P and temperature on D4 as well as D8 GCR (D4GCR *p* = 0.016 and D8GCR *p* = 0.002) and also D4 and D8 gametocytemia (D4GC *p* = 0.004 and D8GC *p* = 0.009). The statistical analysis is summarized in Fig. 2b and the complete analysis is shown in Supplementary data (Supplementary Tables 2 & 3). The final model indicates that there is a positive correlation between D0P and GCR for patients with lower temperatures, but this association decreases as temperature increases. The negative influence of temperature on the association between D0P and GCR is demonstrated graphically by comparing the slopes of the trend lines for the lnD0P verse lnGCR plots for patients with temperatures < or ≥ the median temperature of 38.5 °C (Fig. 2c and d).

### Metabolomic analysis of host plasma

**Fig. 3:**
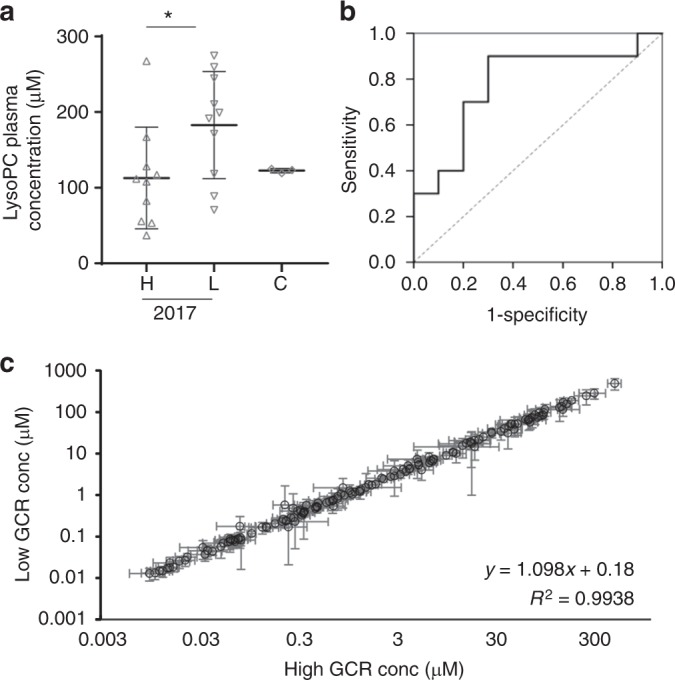
The association of LysoPC level and gametocyte conversion. **a** The total concentration of all 13 LysoPC isoforms tested is plotted for each of the samples in the 2017 high (H) (*n* = 10) and low (L) (*n* = 10) GCR cohorts and control serum from North American volunteers (*n* = 3) (C). Significance was determined using a Mann–Whitney test (*p* = 0.036). **b** Receiver Operating Characteristic curve was constructed for the total LysoPC concentration of each of the 2017 samples as classifiers in the high GCR (*n* = 10) and low GCR (*n* = 10) cohorts. The area under the curve was 0.78 (95% confidence interval 0.57–0.995) (*p* = 0.03). **c** Comparison of the average concentration of each of the 153 nonLysoPC metabolites in the 2017 high GCR (x axis) and the low GCR (y axis) cohorts. For each of the 153 nonLysoPC metabolites detected in the Biocrates AbsoluteIDQ ® p180 analysis the average concentration was calculated for the 10 samples in the 2017 high GCR cohort (High GCR conc) and the 10 samples in the 2017 low GCR cohort (Low GCR conc) and plotted on the *x* and *y* axes, respectively. Each dot represents one of the 153 metabolites ± s.d. The correlation coefficient (*R*^2^) and equation for the trend line are included

Both asexual parasitemia and temperature have been reported to be associated with serum lysophosphatidylcholine (LysoPC) levels in malaria patients^41^ and LysoPC levels have recently been implicated in gametocytogenesis in vitro^27^. To focus the initial analysis, we first compared individuals with high and low to undetectable GCR. Forty plasma samples (Supplementary Table 4) with a range of D0P (>0.35%), 20 (10 from each year) that have consistently high GCR (D4 GCR > 4.9%) and 20 (10 from each year) with low to undetectable GCR were selected for metabolomic analysis using the Biocrates AbsoluteIDQ® p180 kit and liquid chromatography tandem mass spectroscopy (LC-MS/MS). On first analysis there is no significant difference between the mean of the 13 LysoPC isoforms in the high and low GCR samples. However, there is a significant difference between the mean total LysoPC isoform levels in 2016 and 2017 (Mann–Whitney test, *p* = 0.0035) although the mean levels in either year are not different from uninfected control plasma and no other clinical parameters, including parasitemia, WBC, Age, Hb levels or temperature differed between the 2016 and 2017 patients in the cohorts (Supplementary Fig. 2a and b). Interestingly, in the 2017 cohort there is also a significant difference between the total LysoPC levels in the high and low GCR cohorts that is not observed in the 2016 cohort (Fig. 3a, Supplementary Fig. 2A, C&D). Notably, 7 of the 8 samples with total LysoPC levels above a threshold of 170 μM had no detectable gametocyte-committed rings. Receiver operating characteristic analysis of LysoPC levels and D4 GCR in the 2017 high and low GCR cohorts has an area under the curve of 0.780 (*p* = 0.034) suggestive of good overall accuracy (Fig. 3b). An optimal level of LysoPC at 170 µM had a sensitivity of 90% and a predictive false positive rate of 30% for predicting low D4 GCR. When the metabolomics analysis is extended to the remaining 153 metabolites detected in the Biocrates AbsoluteIDQ® p180 analysis no significant differences (FDR < 0.05) are detected between the average concentration of the metabolites in the high and low GCR samples in 2016, 2017 or in the combined data set. When the mean concentrations of the metabolites in the 2017 high and low cohorts are plotted against each other the slope of the line is 1.098 with an *R*^2^ coefficient of 0.994 (Fig. 3c) indicating overall similarity in the two cohorts. Notably, none of the 153 metabolites falls significantly above or below the trend line indicating similar concentrations in both cohorts.

### Early gametocyte gene expression is associated with high GCR

**Fig. 4:**
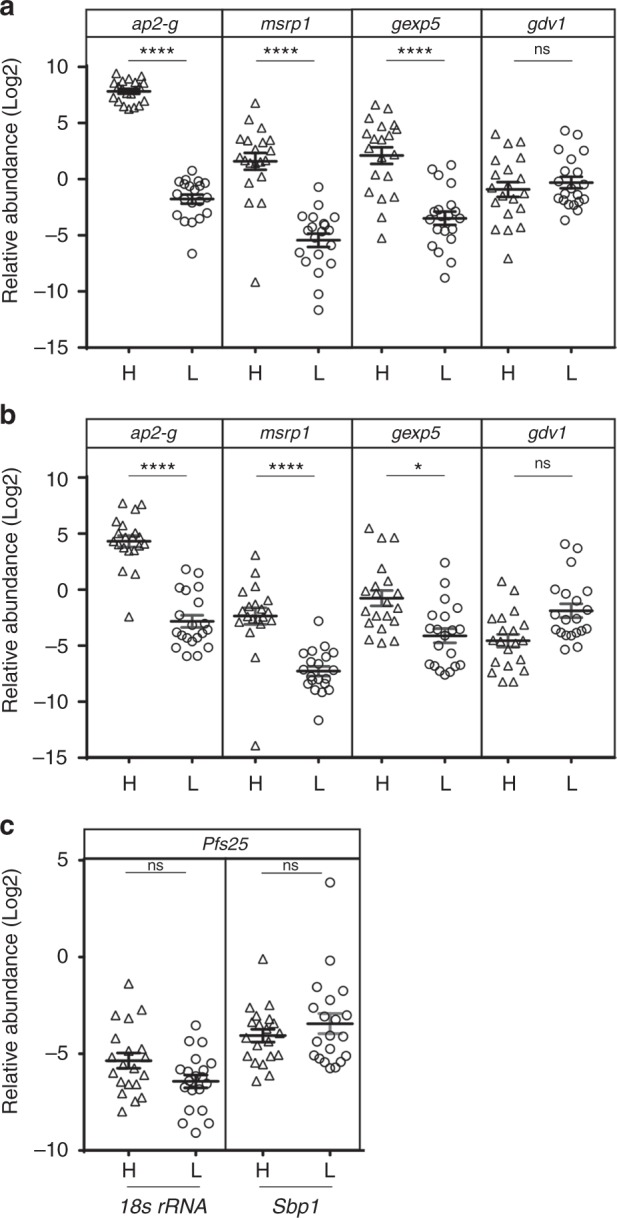
In vivo, high gametocyte conversion is associated with high *ap2*-*g*, *msrp1* and *gexp5* transcript levels. **a**, **b** The relative abundance (2^−ΔΔC^_T_) of the transcript levels of the indicated genes were determined for malaria patient samples in the high GCR (H, triangles, *n* = 20) and low GCR (L, circles, *n* = 20) cohorts using *18s* *rRNA* (**a**) or *sbp1* (**b)** RNA levels as the endogenous control. The reference sample was RNA from *Pfgdv1.gfp.dd*.T1 parasites grown in the absence of Shld1 to inhibit gametocyte production. **c** The relative abundance (2^−ΔΔC^_T_) of *Pfs25* transcripts in the high GCR (H, triangles, *n* = 20) and low GCR (L, circles, *n* = 20) cohorts was determined using *18s* *rRNA* or *sbp1* RNA as the endogenous control and, again, the reference was RNA from *Pfgdv1.gfp.dd*.T1 parasites grown in the absence of Shld1 to inhibit gametocyte production. The experiment was performed in duplicate and the means and s.e.m. are shown. A Kruskal–Wallis test followed a Dunn’s multiple comparison test was used to compare between high and low converters and probability is indicated, *p* > 0.05 (ns), *p* < 0.05 (*) and *p* ≤ 0.0001 (****)

We then sought to evaluate potential in vivo molecular markers for early *P. falciparum* gametocytes in field samples using the same 40 samples used in the metabolomics study that included 20 with high and 20 with low GCR (Supplementary Table 4). In addition to *gdv1* and *ap2-g*, we selected one *ap2-g*-dependent gene, *msrp1*^24^, and one *ap2-g*-independent early gametocyte gene, *gexp5*^35^ to evaluate in the D0 blood samples using *18s rRNA* as well as a ring stage-specific gene, *skeleton-binding protein* (*sbp1*, PF3D7_0501300 [https://plasmodb.org/plasmo/app/record/gene/PF3D7_0501300]) as constitutive controls for parasitemia (Fig. 4a & b). Ring-stage *Pfgdv1.gfp.dd* parasites grown in the absence of Shld1 to inhibit gametocyte production are used as the reference group to calculate relative abundance for the high and low GCR samples. *Ap2-g*, *msrp1*, *gexp5* transcript levels are all significantly higher in the high GCR samples than the low GCR samples using either *18s rRNA* or *Sbp1* as the control, while there is no difference in *gdv1* RNA levels in the two groups. *Pfs25* transcript levels are also tested to evaluate stage V gametocyte levels in the high and low GCR samples and found not to differ (Fig. 4c). This work is the first demonstration that the in vivo expression levels of these gene correlate with ex vivo sexual differentiation, providing support for their use as biomarkers in the field and indicating that the changes in gene expression underlying gametocytogenesis are similar in vivo and in vitro.

### Inducible regulation of gametocyte production

**Fig. 5:**
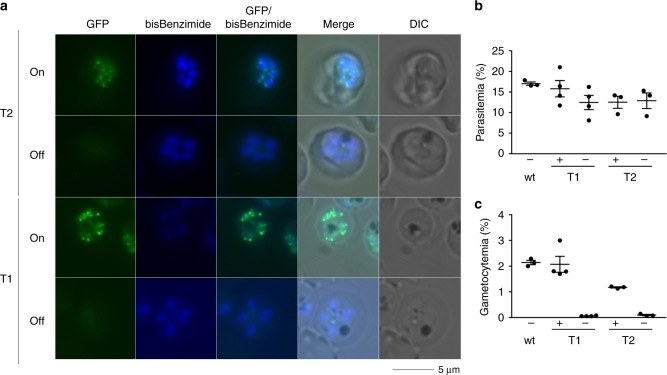
Shld1-dependent GDV1-GFP expression and gametocyte production in *Pfgdv1.gfp.dd* parasites. **a** Representative fluorescence microscopy images of unfixed *Pfgdv1.gfp.dd*.T1 and T2 parasites cultured in the presence (On) or absence (Off) of 500 nM Shld1. *Pfgdv1.gfp.dd* parasites were re-suspended in PBS containing bisbenzimide (5 µg/ml) and visualized using a Zeiss Axiovert 200 fluorescence microscope at ×1000 magnification using AxioVision v4.3.0 101 software. **b**, **c** Shld1-dependent *Pfgdv1.gfp.dd* gametocyte production. Parental NF54 (wt) and *Pfgdv1.gfp.dd* clones T1 and T2 were set up at 1% parasitemia and maintained in the presence ( + ) or absence (−) of 500 nM Shld1 for 12 days. NAG (50 mM) was included in the media days 5–12. Parasitemia as determined using Giemsa-stained smears made on day 5 **a** and day 12 **b**. The mean ± s.e.m. of three independent experiments is plotted

**Fig. 6:**
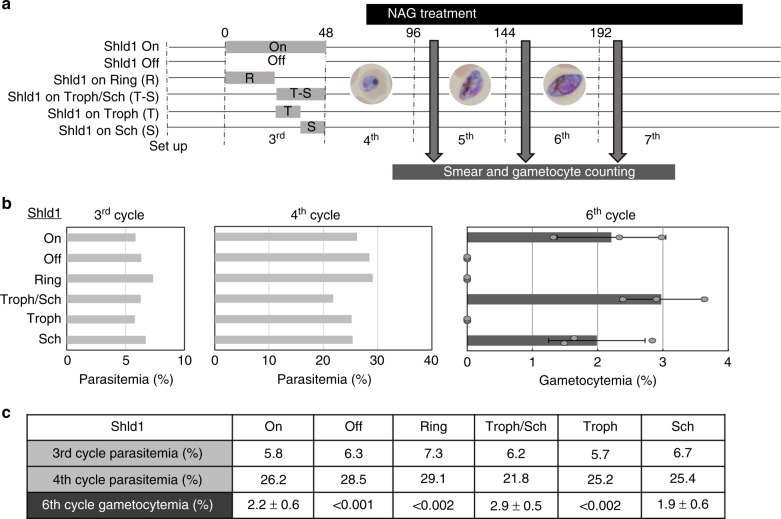
GDV1 expression during schizogony is critical for gametocyte production. **a** Schematic of the experimental design. *Pfgdv1.gfp.dd*.T1 parasites grown in the absence of Shld1 until the parasitemia reached 4–6% rings were divided into 6 wells and Shld1 was added only for the following time intervals: Shld1 On) 1–48 h, Shld1 Off) 0 h, Shld1 on Ring) 0–24 h (R), Shld1 on Troph/Sch) 24–48 h (T-S), Shld1 on Troph) 24–36 h (T), Shld1 on Sch) 36–48 h (S). The following asexual cycle NAG (50 mM) was added at the trophozoite stage (~72 h) and continued for the course of the experiment. Culture smears were made at each cycle to track parasite development and a Giemsa-stained image of the predominant parasite stage is shown. **b** The mean parasitemia for each condition in the 3rd and 4th cycle as well as the mean ± s.d. gametocytemia in the 6^th^ cycle are plotted and the values are listed (**c**). The experiment was repeated twice and the data shown is from one representative experiment

We next wanted to extend our analysis to the regulation of these genes during the transition from schizogony to gc-rings. However, due to sequestration of mature intraerythrocytic asexual parasites, schizonts are not accessible in human peripheral blood and therefore cannot be studied directly in patient samples. Given the similar upregulation of these genes in gc-rings in vivo and in vitro, we decided to begin to study this in vitro using a transgenic parasite line (*Pfgdv1.gfp.dd*) that allowed inducible gametocyte production. In this parasite line the endogenous *gdv1* gene in strain NF54 parasites is tagged with *green fluorescent protein* (*gfp*) followed by a Shld1 ligand-dependent FKBP-derived destabilization domain allowing GDV1 protein-dosing experiments by varying Shld1 concentrations. Two independently transformed lines (*Pfgdv1.gfp.dd*. T1 and T2) were tested for chromosomal integration (Supplementary Fig. 3A & B), GFP expression (Fig. 5a), and *gdv1.gfp.dd* transcript (Supplementary Fig. 3C) as well as gametocyte production in the presence and absence of Shld1 (Fig. 5c). As anticipated, *gdv1.gfp.dd* transcript levels are insensitive to Shld1, while Shld1 is required for GFP expression confirming successful integration into the *gdv1* locus and Shld1-dependent GDV1.GFP.DD expression. The perinuclear pattern of GFP expression in schizonts is the same as that observed previously in parasites transformed with a plasmid directing episomal expression of GFP- or HA epitope-tagged GDV1^24^ demonstrating that protein localization is not affected by the addition of the DD at the C-terminus. GFP expression is first observed as DNA replication began, ~36 h post invasion, which is consistent with peak *gdv1* transcript levels (www.PlasmoDB.org^42^)^43^ (Supplementary Fig. 4). The GFP signal is observed by microscopy in all schizonts and remained high for 8 h before dissipating during merozoite segmentation and GFP expression is not observed in the newly formed ring stage parasites. Schizogony is monitored by flow cytometry using DNA stain SYTO 59, but the GDV1.GFP signal is not strong enough to be detected above the background green autofluorescence that also increased during schizogony. As expected Shld1 stabilization of GDV1.GFP.DD is also required for wild type levels of gametocyte production in both *Pfgdv1.gfp.dd* clonal lines (Fig. 5b & c). In the absence of Shld1 only an occasional gametocyte is observed, while exposure to Shld1 for only the last 12 h of the asexual cycle (36–48 h post RBC invasion) is sufficient to generate merozoites that are fully competent to invade red blood cells (RBCs) and differentiate over the next 10–12 days through the 5 morphologically distinct stages of gametocyte development (I–V) (Fig. 6).

### GDV1 regulates *ap2-g* as well as *ap2-g*-independent genes

**Fig. 7:**
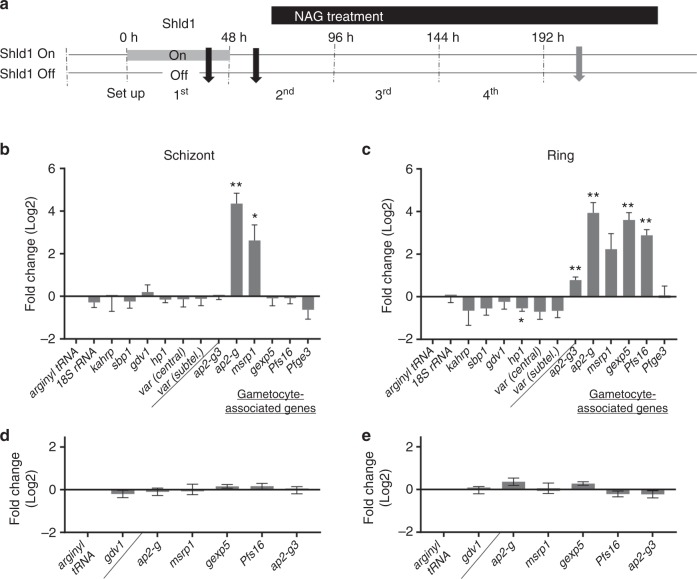
GDV1-dependent increase in gametocyte-associated genes in *Pfgdv1.gfp.dd*.T1 parasites. **a** Schematic of the experiment indicating the Shld1 treatment time. The RNA collection times are indicated by black arrows (38 h Schizonts and 24 h later during the subsequent ring stage) and the grey arrow designates the time the Giemsa-stained smear was made to assess gametocyte production. **b**–**e** The fold change in the expression of each gene (2^−ΔΔC^_T_) in *Pfgdv1.gfp.dd*.T1 (**b**, **c**) or wild type NF54 (**d**, **e**) parasites at schizont- (**b**, **d**) or ring- (**c**, **e**) stages in the presence and absence of Shld1 is shown. The Shld1 minus schizont stage value for each gene is used as a reference and the mean ± s.e.m. of triplicate or quadruplicate flasks from three independent experiments is plotted. A two-way ANOVA with Sidak’s multiple comparisons test followed by a Bonferroni post-hoc test to correct for the analysis of 13 genes was used to determine significance and probability is indicated, *p* ≤ 0.05 (*), *p* ≤ 0.01 (**). The individual 2^−ΔΔC^_T_ for each gene are shown in Supplementary Fig. 5

The ability to control gametocyte production in a clonal parasite line using Shld1 allowed direct analysis of the initial stages of sexual differentiation without contaminating subpopulations of older gametocytes present in wild type *P. falciparum* cultures. The expression of a range of stage-specific and constitutive transcripts were compared in MACS/sorbitol synchronized cultures that were grown in the presence or absence of Shld1 (Fig. 7 and Supplementary Fig. 5). RNA obtained during schizogony [36 ± 2 h post invasion (hpi)] and 24 h later, during the subsequent ring stage, was tested for gametocyte-associated transcripts, *ap2-g*, *msrp1*, *gexp5*, *Pfs16* (PF3D7_0406200)^24,44^ *Pfge3* (PF3D7_1477700)^45^ and *ap2-g3* (PF3D7_1317200)^46,47^, which was reported in a piggyBac screen for gametocytogenesis related genes in *P. falciparum* and has recently been shown to be upstream of *ap2-g* expression in the rodent malaria *P. yoelii*. In addition, *18s rRNA* (PF3D7_0725600) and two asexual ring specific genes, *kahrp* (PF3D7_0202000)^14^ and *sbp1*^48^ were tested as well as *hp1*-specific primers and conserved primers that amplify multiple *var*^31^ genes which are regulated by *hp1* (Supplementary Table 5).

In schizonts from Shld1-treated vs untreated *Pfgdv1.gfp.dd*.T1 cultures we found a significant increase only in mRNA corresponding to *ap2-g*, and *msrp1* (Fig. 7b & Supplementary Fig. 5A), which is not observed in parental NF54 parasites (Fig. 7d and Supplementary Fig. 5B). This Shld1-dependent increase is maintained in RNA harvested 24 h later from the ring stage *Pfgdv1.gfp.dd*.T1 parasites (Fig. 7c and Supplementary Fig. 5A), while there continued to be no Shld1-dependent difference in NF54 parasites (Fig. 7e and Supplementary Fig. 5B). Transcripts for *gexp5* as well as an additional early gametocyte gene, *Pfs16*, are also found to be significantly up-regulated in ring stage parasites from the Shld1-treated *Pfgdv1.gfp.dd*.T1 group. At this early time point *Pfge3* RNA levels are not significantly elevated. As expected there is no Shld1-dependent change in transcript levels for *18s rRNA*, *kahrp*, or *sbp1*, which have not been associated with early gametocytes, but are included as controls for parasite stage. Additionally, RNA levels for *gdv1*, and *var* genes are not affected by Shld1 treatment, indicating that ligand-mediated stabilization of GDV1 is not required for expression of these genes. *Ap2-g3* and *hp1* transcript levels are also not affected by Shld1 treatment during schizogony, but in the subsequent ring stage RNA levels are slightly lower for *hp1* and slightly higher for *ap2-g3*.

**Fig. 8:**
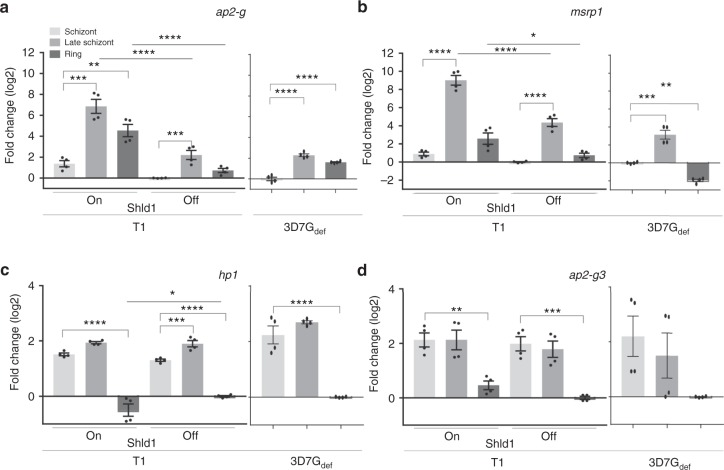
Gene expression patterns in the absence of GDV1. The fold change (2^−ΔΔC^_T_) of *ap2*-*g* **a**, *msrp1* **b** *hp1* **c**, and *ap2*-*g3* **d** RNA levels was tested in early schizonts (36 ± 2 hpi) (light grey), late schizonts (46 ± 2 hpi) (medium grey) and 12 ± 2 h later in ring stage parasites (dark grey) in *Pfgdv1.gfp.dd*.T1 parasites in the absence (Off) and presence (On) of Shld1 (**a**–**d**) as well as the clonal 3D7.G_def_ parasite line that lacks the *gdv1* locus due to a spontaneous chromosome 9 deletion^24^. Expression levels in early schizonts in the absence of Shld1 are used as the reference for *ap2-g* **a** and *msrp1* **b**, while the Shld1 minus ring stage expression levels were used for *hp1* **c** and ap2-g3 **d**. The data are from 2 independent experiments performed in duplicate. To compare expression between stages and conditions in the *Pfgdv1.gf.dd* parasite line a two-way ANOVA with Sidak’s multiple comparisons test was used. For the 3D7.G_def_ line, a one-way ANOVA with Dunnett’s multiple comparisons test was performed to compare expression between stages. For both analyses a Bonferroni post-hoc test to correct for the analysis of 4 genes was used and probability is indicated, *p* ≤ 0.05 (*), *p* ≤ 0.01 (**), *p* ≤ 0.001 (***)

The expression of *ap2-g*, *msrp1*, *hp1* and *ap-g3* in *Pfgdv1.gfp.dd*.T1 parasites was further investigated by isolating RNA from schizonts when *gdv1* transcript levels are increasing (~1–3 nuclei, 36 ± 2 hpi) and then again 10 h later during late schizogony when *ap2-g* levels increase and during the subsequent ring stage another 14 h later. Consistent with the previous experiment, in the presence of Shld1 transcript levels for *ap2-g* and *msrp1* levels increase while in rings *hp1* transcripts decrease and *ap2-g3* transcripts increase slightly (Fig. 8a–d). Notably, even in the absence of Shld1 and in a gametocyte-deficient line (3D7G_def_) that lacks the *gdv1* locus^24^ *ap2-g* and *msrp1* transcript levels increase significantly during schizogony suggesting that GDV1 is not required to initiate transcription (Fig. 8a–b)

**Fig. 9:**
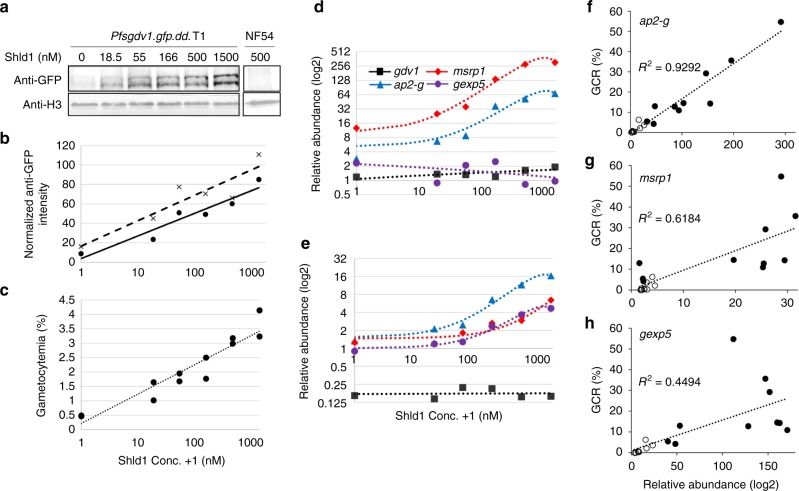
Shld1 dose-dependent increase in GDV1 protein levels. **a** Immunoblot of saponin-treated *Pfgdv1.gfp.dd* or wild type NF54 schizonts extracted in 8 M urea/5% SDS after 24 h incubation in the indicated amount of Shld1. The immunoblot was first probed with anti-GFP mAb and imaged, then reprobed with rabbit anti-Histone 3 (anti-H3) antibody as a loading control as described in Methods. The anti-GFP and anti-H3 positive bands are shown and the complete gel is shown in Supplementary Fig. 6A. **b** The density of the upper (dot) and lower (x) bands was determined after background subtraction using Image J software, normalized to the anti-histone 3 antibody signal and plotted. **c**–**e** Shld1 dose-dependent increase in gametocyte production and *ap2-g*, *msrp1* & *gexp5* transcription. **c** After growth in the indicated Shld1 concentration for ~50 h, NAG was added and gametocyte production (average ± s.d.) was determined 9 days later by counting Giemsa-stained smears. **d,** **e** The average relative abundance (2^−ΔΔC^_T_) of *ap2*-*g* (blue), *msrp1* (red), *gexp5* (purple) and *gdv1* (black) transcripts in RNA obtained from schizonts (46 ± 2 hpi) **d** and 14 h later from ring stage parasites (**e**) grown in the indicated concentration of Shld1 are plotted using early schizonts (36 ± 2 hpi) as the reference. The experiment was run twice with duplicate flasks and a representative data set is shown with the trend lines indicated. The second experiment is shown in Supplementary Fig. 6B-D. **f**–**h** Gametocyte conversion rate is positively correlated with *ap2*-*g*, *msrp1*, *gexp5* mRNA expression. The gametocyte conversion rate and the relative abundance (2^−ΔΔC^_T_) of **f** *ap2-g*, **g** *msrp1* or **h** *gexp5* RNA in ring stage (14 ± 2 hpi) *Pfgdv1.gfp.dd* clone T1 parasites cultured in the absence (open circle) or presence (black dot) of 500 nM Shld1 are plotted. The data are from 3 independent experiments performed in triplicate or quadruplicate. The trend line and correlation coefficient (*R*^2^) is shown

Next we performed a GDV1 protein-dosing experiment by varying Shld1 concentrations (0–1500 nM) to evaluate the role of GDV1 protein levels on *ap2-g*, *msrp1* and *gexp5* expression levels. An anti-GFP monoclonal antibody (mAb) is used to assess GDV1 levels in *Pfgdv1.gfp.dd* schizonts following 14 h treatment with different Shld1 concentrations and demonstrates a dose dependent increase (Fig. 9a & b). GDV1.GFP.DD migrates as two bands and can only be extracted by adding 8 M Urea and 5% SDS, indicating it tightly associates with an insoluble complex, possibly the nuclear envelop. The estimated molecular weight of the anti-GFP positive upper bands from 5 independent experiments is 113 ± 2.8 kDa (mean ± s.d.), which is consistent with the predicted molecular weight of the full length chimeric protein (111.8 kDa) suggesting the lower band (100 ± 1.7 kDa) is processed, but this needs to be further tested.

To test for a dose-dependent effect of Shld1 on transcript levels, RNA was collected from late schizonts (46 ± 2 hpi) and 14 h later from the resulting ring stage parasites. Transcripts for *ap2-g*, *msrp1* and *gexp5* genes, as well as gametocyte production (Fig. 9c–e & Supplementary Fig. 6) increase in a dose dependent manner with a plateau in RNA levels at the concentration of Shld1 normally used to stabilize GDV1 in culture (500 nM). The similar Shld1 dose response curve for gametocyte production and RNA levels suggests that these transcripts might be potential markers for early gametocytes. We test this correlation directly using the data obtained over the course of our various different Shld1 experiments (Fig. 9f–h). The *R*^2^ values for correlation between gametocyte conversion and the RNA levels of each of the 3 genes and are > 0.44 in ring stage parasites. In particular, *ap2-g* levels in ring stage parasites and gametocyte conversion have an *R*^2^ of 0.92. In total, these data suggest the capacity of GDV1 protein levels to modulate gametocyte formation and transcription levels of downstream genes.

### *Gdv1* allele frequency in high and low GCR samples

**Fig. 10:**
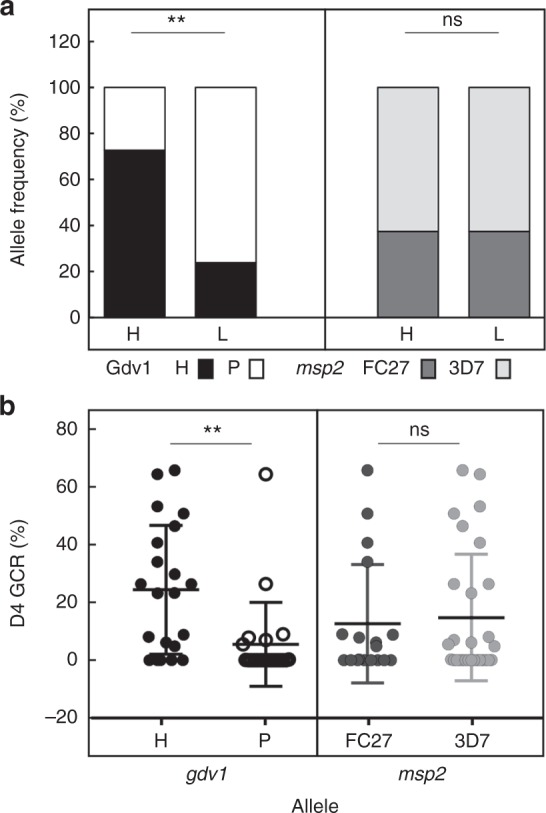
GDV1 allele H^217^ is over represented in high GCR patient samples. **a** The frequency of the *gdv1* alleles encoding H (black) and P (white) or (B) *msp2* FC27 (dark grey) and 3D7 (light grey) family alleles in the high (H) (*n* = 20) and low (L) GCR (*n* = 19) samples are plotted. Significance was assessed using a Fisher Exact test. **b** The D4 GCR (%) of each of the samples with *gdv1* allele H^217^ (H, black dot) or P^217^ (P, open circle) or *msp2* allele FC27 (dark grey dot) or 3D7 (light grey dot) is plotted with the mean and s.d. indicated. The Kruskal–Wallis test followed a Dunn’s multiple comparison test was used to compare the D4 GCR of the samples with distinct *gdv1* or *msp2* alleles (Prism GraphPad v7.05). Probability is indicated, *p* > 0.05 (ns) and *p* ≤ 0.01(**)

The ability of GDV1 to act as a rheostat for gametocyte production coupled with prior field work that identified a SNP in *gdv1* (Chr9:1378602)^29^ whose population frequency varied with transmission intensity prompted us to evaluate this SNP in patient samples in the high and low GCR cohorts that were used for metabolic analysis (Supplementary Table 4). Dried blood spots for DNA analysis were available for all twenty 2017 samples and all but one of the twenty 2016 samples. The high and low GCR samples have significantly different *gdv1* allele distributions (Fisher exact test, p = 0.022) (Fig. 10), while there is no difference in *msp2* allele frequencies in the two cohorts (Fig. 10). The frequency of the H^217^ GDV1 allele previously associated with a limited seasonal malaria in The Gambia is higher in the high GCR samples (n = 20, 73%), while the frequency of the P^217^ allele previously associated with continual malaria transmission in the Republic of Guinea is higher in the low GCR cohort (n = 19, 76%). This finding suggests that in areas with limited seasonal transmission H^217^ parasites may be selected for the ability to produce a higher ratio of gametocyte- to asexually committed rings to maximize transmission.

## Discussion

Here we report the first direct comparison of the presence of gametocyte-committed ring stage parasites in human blood samples with clinical parameters and parasite transcript levels. The GCA allowed detection and quantification of gametocyte generating capacity 10 days before the circulation of transmissible stage V gametocytes. The results indicate that gc-rings circulate in most patient’s peripheral blood, however the levels vary dramatically, from 78% to no detectable sexually committed rings. This pattern is consistent with a low basal level of sexual differentiation that is modulated by D0 parasitemia, patient temperature, *gdv1* allele, GDV1-dependent gene expression and, possibly, LysoPC levels. The GDV1-dependent genes are also strong candidates for sensitive molecular markers that could be used to identify symptomatic and asymptomatic gc-ring carriers to ensure treatment before the production of circulating transmissible stage V gametocytes.

The GCA assay developed in this work is a valuable first step toward evaluating gametocytogenesis in the field. The strong concordance of the GCR values calculated in the 2016 and 2017 malaria seasons as well as on days 4 and 8 demonstrate the reproducibility of the gametocyte counts and the ability of the stage II/III gametocytes detected on D4 to continue to mature to D8. The results are consistent with the circulation of gc-rings prior to sequestration in the bone marrow^49^. Although we cannot rule out a subpopulation of sequestered gc-rings that never circulate, the circulating gc-rings provide a window to assess gametocytogenesis in blood samples within 24 h of the production of gametocyte-committed merozoites. This early detection of gc-rings allows much closer association with host and clinical parameters that may contribute to the balance between asexual and sexual differentiation. The further development of sensitive molecular gc-ring markers coupled with the existing stage V gametocyte markers (*Pfs25* and *mget*, Pf3D7_1469900)^50^, should make it possible to track gametocyte maturation over the next 10–12 day in asymptomatic parasite carriers. Such longitudinal analysis was not possible in this study of symptomatic malaria patients because they had to be treated. At enrollment most of the study population had RT-qPCR detectable circulating stage V gametocytes as well as gc-rings and there was a wide range of levels for both stages. However, there was no significant correlation between the number of stage V gametocytes in a D0 sample and its GCR suggesting that 10–12 days earlier their GCR was different. As mentioned above the only way to directly study the relationship between GCR and stage V gametocyte production is a longitudinal study monitoring asymptomatic individuals for the progression of gc-rings to stage V gametocytes two weeks later. We anticipate that young children at the beginning of the malaria season before the development of immunity would have a direct correlation between gc-ring markers/GCR, and stage V gametocytes 2 weeks later, which would provide in vivo validation for the GCR assay and gc-ring markers.

The positive association we found between D0P and GCR is consistent with previous in vitro data demonstrating that sexual differentiation increases with high parasitemia^51,52^, but attempts to identity a consistent density dependent trigger have been challenging^21^. Recently, LysoPC-depleted culture media has been shown to increase conversion rate in vitro^27^, but was not examined in humans. In the 40 samples we tested, the total plasma LysoPC levels ranged between (40–275 μM) and varied significantly between collection years. In 2017, high LysoPC levels are associated with low GCR, which is consistent with high plasma LysoPC having a negative influence on gametocyte production. The reason for the difference in LysoPC levels in the 2016 and 2017 plasma samples is unclear. As there was no significant difference in the other clinical parameters between the two years, it is possible that different plasma storage conditions in 2016 and 2017 could have been responsible. However, LysoPC levels have also been shown to be influenced by body weight^53,54^ and infection; decreasing during severe malaria^41^, African trypanosomiasis^55^, and sepsis^56^ so alternative explanations cannot be ruled out completely.

In contrast to low D0 parasitemia^51^ and high LysoPC levels^27^, high temperatures have not previously been specifically associated with a decrease in sexual differentiation^57^, but have been shown to decrease parasite growth and viability^58,59^. The negative influence of high D0 temperature on the positive relationship between D0 parasitemia and GCR was consistently observed on D4 and D8, but the effect was more pronounced on D4. This difference in the influence of temperature could be due to a temperature-induced delay in growth decreasing the number of gametocytes that reached stage II by D4. Stage II is the first morphologically distinct gametocyte stage so a delay would reduce the D4 gametocyte count, but could have less effect on the D8 counts which included stages III–V. An inverse relationship of D0 parasitemia and fever on GCR suggests that for a given parasitemia gametocyte production would be higher in asymptomatic individuals, which is consistent with recent work showing a positive correlation between parasite density and stage V gametocytes in asymptomatic infections^50^ and a negative correlation in a meta-analysis of symptomatic cases^40^. This inverse relationship between D0P and temperature also suggests that sexual differentiation would increase as an individual’s pyrogenic threshold to *Plasmodium* infection increases^39^ and this may contribute to the relative increase in mature gametocytes to asexual parasites that has been observed with age^16^.

In addition to the negative effect of temperature on sexual differentiation, another unexpected finding was the lack of correlation between high GCR and low hematocrit. Low hematocrit has previously been reported to be associated with an increase in circulating stage V gametocytes in patient blood smears^15,17,18^. The lack of correlation between GCR and hematocrit suggests that low hematocrit is not the major independent driver of sexual differentiation in this study. It is possible that the low hematocrit previously associated with circulating stage V gametocytes could have been due to the prolonged infection required for enough gametocytes to mature 10 days and be released into the circulation as stage Vs for detection by Giemsa-stained blood smear. Such differences between the factors associated with gc-rings and circulating stage V gametocytes highlight the need to evaluate sexual differentiation longitudinally at 2 week intervals in asymptomatic individuals to assess both gc-rings and the resulting stage V gametocytes that circulate 2 weeks later.

Sexual differentiation in vivo was also associated with an increase in RNA levels of three genes shown to be dependent on GDV1 protein levels in vitro, as well as the H^217^ GDV1 allele previously reported to be over represented in a region with limited seasonal malaria transmission^29^. Together, this data is consistent with GDV1 playing a key role in gametocytogenesis in vivo and the H^217^ GDV1 allele inducing sexual differentiation more effectively than the P^217^ allele in the field. In contrast to histidine, proline restricts the flexibility of the peptide chain and could alter protein structure potentially affecting function^60^, but this needs to be further evaluated by more detailed genetic studies. A recent field study extending the analysis of the *gdv1* locus to additional West Africa locations found a structural dimorphism downstream from the *gdv1* coding region that also demonstrated strong geographical divergence, but was not clearly linked to transmission intensity^61^. In contrast, an association between low malaria transmission and high expression levels of gametocyte-associated genes, including *ap2-g*, has recently been reported in a publication comparing the transcriptomes of parasites isolated in areas with different patterns of malaria transmission^62^. However, they did not directly assess gametocyte production or *gdv1* allele. Clearly further work is needed to integrate these intriguing findings.

To extend our molecular analysis of the regulation of gametocytogenesis, we used *Pfgdv1.gfp.dd* parasites that allow inducible gametocyte production by Shld1-dependent protection of GDV1 expression. GDV1 was required only during schizogony for the induction of gametocytes. Since schizonts are sequestered in vivo and not present in human blood samples we assessed regulation in vitro and found *ap2-g* and *msrp1*, not *gexp5*, transcript level were significantly increased during schizogony in the presence of GDV1. However, close analysis of the in vitro data during schizogony in the *Pfgdv1.gdf.dd* line and a *gdv1* deficient line (G_def_) indicates that, although *ap2-g* RNA levels are lower in the absence of GDV1, *ap2-g* transcripts still increase significantly during the transition from early to late schizonts. This low but significant GDV1-independent increase in *ap2-g* transcript levels suggests GDV1 is required to augment *ap2-g* mRNA levels during schizogony, rather than being directly involved in initiating transcription or required to release repression by displacing or preventing HP1 binding to H3K9Me3. The ability of Shld1-mediated stabilization of GDV1 to enhance *ap2-g* RNA levels not *var* gene expression, both of which are repressed by HP1^31^, also argues against a direct effect of GDV1 on all HP1 repression sites. Based on recent data from *P. yoelii* it is possible that another *ap2* transcription factor, *ap2-g3* is required for this initial *ap2-g* expression and here we show that *ap2-g3* levels are not affected during schizogony by GDV1 levels. It is possible that *ap2-g3* initiates *ap2-g* transcription, while GDV1 is required to augment transcription or stabilize RNA levels, which is an established regulatory mechanism in *P. falciparum*^63–65^. Future functional studies will include clinically relevant parasitemias, temperatures, LysoPC levels and *gdv1* alleles in vitro to better define the signals influencing gametocyte production.

One of the major limitations of this study is that all the volunteers were recruited from a single clinic in an area of high seasonal transmission with a low levels of microscopically detectable stage V gametocytes^66^. Reproducibility in different regions with different malaria transmission patterns and a wider range of ages is needed to validate the clinical parameters and gene expression profiles found to be associated with gc-ring stage parasites. To extend the study to include lower parasitemias and remote areas without access to tissue culture facilities or trained staff to maintain and quantify the ex vivo cultures, robust molecular markers are needed to identify gc-rings. Such markers will also allow the direct comparison of gc-ring production and maturation to stage V gametocytes two weeks later.

In summary, circulating gc-rings were detected in 76% of the 260 malaria patients tested using the GCA developed here. The ratio of gametocyte- to asexual-committed rings varied widely and was associated positively with D0 parasitemia and negatively influenced by patient temperature. GDV1 allele H^217^ and transcript levels of three GDV1-regulated genes, *ap2-g*, *msrp1* and *gexp5* were significantly higher in individuals with high ratios of gametocyte- to asexual-committed rings, while in the 2017 plasma samples high levels of LysoPC were associated with low levels of gametocyte production. In vitro, GDV1 was found to play a critical role in the regulation of the balance between asexual and sexual development during schizogony, prior to the production of gametocyte-committed rings. The combined findings support consistent low-level gametocytogenesis in the human host that is modulated by parasitemia and fever as well as GDV1 levels and allele.

## Methods

### Study site and Population

Blood samples were collected from children attending the Ewim Health Center in Cape Coast, Ghana^66^. Briefly, children ( ≤ 13 yrs) with *P. falciparum* parasitemia ranging between 1,000 and 250,000 per µl of blood based on the WHO standardized protocol^67^ were recruited into the study and their axillary temperature recorded.

### Ethics Statement

The study was approved by the Institutional Review Board of the Noguchi Memorial Institute for Medical Research and the Ghana Health Services and reviewed by DMID, NIAID, NIH. Before recruitment each parent/guardian was informed of the objectives, methods, anticipated benefits and potential hazards of the study. The parents/guardians were encouraged to ask questions about any aspect of the study that was unclear to them and informed about their liberty to withdraw their children at any time without penalty. Children were enrolled only after written parental consent had been obtained. All patient information is treated as confidential.

### Sample collection

Blood samples were obtained from consented children prior to drug treatment (D0). For each subject, blood was collected into acid citrate-dextrose solution (ACD) (2.0 ml) for parasite cultures, a tube containing ethylenediaminetetraacetic acid (EDTA) (0.5 ml) (BD Biosciences, San Jose, CA) for hematological analysis and in 2016 a PAXgene RNA blood tube (2.5 ml) (BD Biosciences) for RNA isolation. A Urit 3000 Plus (Urit Medical Electronics, Guilin, Guangxi 541004.P.R.China) was used to assess the hematological parameters, including the WBC count and Urit-12 hemoglobin meter (Urit Medical Electronics) was used to determine hemoglobin levels according to manufacturers’ instructions. Immediately after removing the samples for the hematological indices and making a thick blood smear, the EDTA sample was centrifuged and the plasma stored at −20 °C for immunological studies. The thick blood smear was stained with Giemsa and used to quantify asexual and sexual stage parasites against 200 WBCs. In 2017 the cell pellet was resuspended in NucleoZOL and stored frozen until RNA analysis. All study participants were given a standard curative dose of artemether-lumefantrine (20/120 mg/kg) or artesunate-amodiaquine (4/10 mg/kg) and scheduled for a follow up visit 7 days later.

### Ex vivo analysis of gametocyte commitment

The samples collected in ACD tubes were centrifuged for five minutes at 2000 rpm to harvest plasma. The cells were washed twice with 5 ml of sterile RPMI 1640 media without serum or AlbuMAX II (Thermo Fisher Scientific, Waltham, MA) and centrifuged at 2000 rpm for five minutes to remove white blood cells and the buffy coat. The red blood cell pellet was resuspended to a 3% hematocrit in RPMI 1640 media supplemented with 2% inactivated human serum and 0.5% AlbuMAX II. Aliquots of the resuspended RBCs (3 ml) were added into 2 separate wells of a 12 well plate. NAG was added and then the plate was incubated at 37 °C in an atmosphere of 5% CO2/5% O_2_/90% N_2_ for 8 days with daily media changes and thin smear preparation but without RBC supplementation. On the 8^th^ day, the cultures were harvested and the parasites preserved in NucleoZOL (Macherey–Nagel). D0, D4 and D8 parasite thin smears were Giemsa-stained and the distinct parasite stages observed by microscope (×1000) were counted. A total of 2000 RBCs were counted to determine the D0 ring stage parasitemia, while 20,000–30,000 RBCs were counted per smear from each of the duplicate D4 and D8 cultures. A sample was classified as gametocyte-deficient if no gametocyte was observed in 30,000 RBCs. Gametocyte conversion rates were calculated for each sample by dividing the average D4 stage II–III gametocytemia or the average D8 stage III–V gametocytemia from the two NAG-treated ex vivo cultures by the D0 parasitemia. For quantitative reverse transcriptase-polymerase chain reaction (RT-qPCR) analysis of the D0 blood sample, RNA was purified using the PAXgene RNA kit (Qiagen, Hilden, Germany) according to the manufacture’s protocol and converted to cDNA for RT-qPCR as described below in the *Quantitative reverse transcriptase-polymerase chain reaction* section. The gene specific primers were designed using Primer3 version 4.1.0 and are listed in Supplementary Table 5 and the 2^−ΔΔC^_T_ for each gene was calculated using *18s rRNA* or *sbp1* as the endogenous control and RNA levels in ring stage *Pfgdv1.gfp.dd*.T1 parasites grown in the absence of Shld1 as the reference. To quantify stage V gametocytes, *Pfs25* RNA levels were determined using RT-qPCR with *Pfs25* specific primers. As a positive control uninfected human blood was mixed with purified NF54 stage V gametocytes and then added to PAXgene tubes or NucleoZOL to replicate the RNA isolation described above for the 2016 or 2017 samples. Stage V parasite density per microliter was calculated using a hemocytometer to quantify the cell concentration and Giemsa-stained thin smears to determine the stage V gametocytemia. The control RNA was serially diluted and used as a RT-qPCR template to generate a standard curve for *Pfs25* (Supplementary Fig. 1). Using the slope of the standard curve to convert *Pfs25* C_T_ to stage V parasites, we then calculated the number of stage V gametocytes per microliter of blood in each sample (Fig. 1f). Similarly, to quantify *Pfs25* transcript levels, a region of *Pfs25* (24–128 bp) containing the RT-qPCR site was PCR amplified (primers PF3D7_1031000-Fw to PF3D7_1031000–1RvCL, Supplementary Table 5), inserted into a Topo-TA plasmid and used to transform chemically competent One Shot® TOPO *E. coli*. A single colony was isolated, the plasmid purified, and the insertion of a single copy of *Pfs25* confirmed by sequencing. The plasmid concentration was quantified by absorbance at 260 using a Qubit 3.0 Fluorometer (Thermo Fisher Scientific) and the plasmid copy number per microliter was determined using an online web-tool (https://cels.uri.edu/gsc/cndna.html). The plasmid was serial diluted and used as a qPCR template to generate a standard curve correlating *Pfs25* C_T_ to transcript number. The results were used to calculate the number of *Pfs25* transcripts per microliter of blood in each sample (Fig. 1f). The number *Pfs25* transcripts/µl was then divided by the number of ring stage parasites per microliter of blood measured from the Giemsa-stained D0 thick smear to determine the % sexual stage parasitemia (Fig. 1g).

### Plasma metabolomic analysis

Plasma from 40 samples with D0 parasitemia > 0.35%, 10 with high GCR (5–66%) and 10 with undetectable to low GCR (0–0.3%) from each year (Supplementary Table 4) and three uninfected control samples were selected for evaluation using the Absolute*IDQ* p180 assay (Biocrates, Inc., Washington, DC) to quantify 180 metabolites according to the manufacturer’s protocol at the Duke University’s Proteomic Core Facility. The metabolites are detected using a using a triple quadrupole tandem mass spectrometer and included staple isotope labeled internal standards. Acylcarnitines, glycerophospholipids, and sphingolipids are analyzed by introducing the sample using a Flow Injection Analysis method with a single point calibration to determine concentration. Amino acids and biogenic amines were analyzed by performing by ultra-high pressure LC-MS/MS using a reversed phase analytical column for analyte separation. A seven point calibration curve was used to quantify the amino acids and biogenic amines.

### *P. falciparum* parasite culture

*P. falciparum* strain NF54 was obtained through BEI Resources (MRA-1000 (Patient Line E), contributed by Megan G. Dowler). Briefly, NF54 strain parasites were maintained in an atmosphere of N_2_/CO_2_/O_2_: 90/5/5 and complete RPMI medium containing RPMI 1640, 25 mM HEPES, 50 μg ml^−1^ of hypoxanthine, and 0.3 mgml^−1^ of glutamine (KD Biomedical, Columbia, MD) supplemented with 25 mM NaHCO3 (pH 7.3), 5 μgml^−1^ of gentamicin, and 10% human serum (Interstate Blood Bank, Memphis, TN)^68^. Sorbitol treatment (5%, 10–30 min at 37 °C) and/or MACS® LS columns (Miltenyi Biotec, Auburn, CA) were used for synchronization and parasite stages were quantitated using Giemsa-stained culture smears to determine parasitemia and total RBC.

### Parasite transformations

*Gdv1* was tagged in frame with green fluorescent protein (GFP) followed by the FKBP-destabilization domain (DD) to allow ligand regulated protein degradation and track protein expression. To generate the transformation vector the 3’ end of *gdv1* was amplified using polymerase chain reaction (PCR) and synthetic oligonucleotides corresponding to *gdv1* bp 901–930 with a 5’ *Xho1* site and *gdv1* bp 1762–1797 flanked by *AvrII* (Supplementary Table 5). The PCR amplicon and p1605-GFP-FKBP-int plasmid^69^ were digested with *Xho1* and *AvrII*. Prior to ligation with T4 ligase the digested plasmid was treated with alkaline phosphatase for 1 h. Electrocompetent DH10B *E. coli* were transformed with the ligation reaction and grown overnight on LB plates containing ampicillin (100 µg/ml). Ampicillin-resistant colonies were selected, screened for insert by restriction enzyme digestion and sequenced before being used for parasite transformation. Established protocols^70^ were used for transformation (100–150 µg plasmid DNA per transformation) and WR99210 (2.5 nM)-resistant parasites were obtained from two independent transformations. After 3 weeks off drug, WR99210 (Jacobus Pharmaceuticals, Plainsboro, NJ) was reapplied and the parasites were cloned by limiting dilution. The presence of a single-crossover chromosomal integration was assessed by PCR amplification using primers listed in Supplementary Table 5.

### Imaging

Cells were pelleted, resuspended in an equal volume of phosphate-buffered saline (PBS) and incubated with bisbenzimide fluorescent dye (5 µg/ml) (Thermo Fisher Scientific) for 10 min. The samples (6 µl) were applied to a large glass cover slides (22 × 50 #1), covered with a circular cover slide (12Cir #1), and immediately visualized using a Zeiss Axiovert 200 fluorescence microscope at 1000x magnification using AxioVision software v4.3.0.

### Flow cytometric analysis

Cells were resuspended at 0.01% hematocrit (~1,000 cell/µl) in buffer A (154 mM NaCl, 9.27 mM glucose, 10 mM Tris-HCl, pH 7.4) with 500 nM SYTO 59 and incubated at RT in the dark for 20 to 30 min prior to analysis on an Accuri C6 flow cytometer (BD Biosciences, Franklin Lakes, NJ). The forward and side scatter signals were used to select the total RBC population and then the SYTO 59 signal (Ex 640 nm/Em 675 ± 25 nm) was used to identify *P. falciparum* infected RBCs and monitor DNA replication during asexual development.

### Quantitative reverse transcriptase-polymerase chain reaction

Samples collected at the indicated developmental stages were preserved in NucleoZOL (Macherey–Nagel) and RNA was isolated from the aqueous phase using the RNeasy Micro kit (Qiagen) according to the manufacturer’s instructions. In addition to DNase treatment during RNA isolation on the Qiagen micro columns, purified RNA (1 µg) was treated with gDNA Wipeout Buffer before conversion to cDNA using SuperScript® VILO™ (Thermo Fisher Scientific). Reverse transcriptase minus controls were used to confirm the absence of genomic DNA. cDNA was used as a template for RT-qPCR (QuantStudio3 with Qcapture software 2.9.13, Applied Biosystems, Foster City, CA) with the indicated primers (Supplementary Table 5) and Fast SYBR Green PCR Master Mix (Applied Biosystems) using the following conditions, 20 sec activation at 95 °C, 40 cycles of 1 sec at 95 °C and 20 sec at 60 °C. All samples were run in triplicate and tested for both the gene of interest and the control constitutive gene, *arginyl-tRNA synthetase* (PF3D7_1218600)^31^, on the same plate. The results were analyzed using QuantStudio^TM^ Design & Analysis Software (Applied Biosystems) and the ΔC_T_ values determined by subtracting the mean cycle threshold (C_T_) value for *arginyl-tRNA synthetase* from the mean target gene C_T_. To compare gene expression profiles, the relative quantity (2^−ΔΔC^_T_) was calculated for each gene using RNA from *Pfgdv1.gfp.dd*.T1 parasites grown in the absence of Shld1 as the reference. The efficiency of the primers was tested by serial dilution and ranged from 87 to 111%.

### GDV1 protein-dosing

*Pfgdv1.gfp.dd* parasite were MACs/sorbitol synchronized and Shld1 concentrations ranging from 0 to 1500 nM added at ring stage. For the immunoblot schizonts harvested 14 h later, treated with 0.05% saponin to remove the RBCs and then extracted in 8 M urea/5% SDS. The immunoblot was probed with anti-GFP mAb (#11-814-460-001, Roche, Indianapolis, IN) followed by horseradish peroxidase-labeled anti-mouse Ig and visualized using the Pierce SuperSignal West Dura kit (Thermo Fisher Scientific) and an ImageQuant LAS 4000 (GE Healthcare, Piscataway, NJ). The blot was then reprobed with rabbit anti-Histone 3 (anti-H3) antibody (#Ab1791, Abcam, Cambridge, MA), followed by alkaline phosphatase labeled secondary Ig and visualized using 5-bromo-4-chloro-3-indolyl-phosphate (BCIP) and nitro blue tetrazolium (NBT) (Thermo Fisher Scientific). The density of the upper and lower bands was determined after background subtraction and normalization to the anti-histone 3 antibody signal using Image J software. For expression profiling, Shld1 was added to ring stage parasites and at 46 ± 2 hpi they were harvested by centrifugation and resuspended in NucleoZOL for RNA preparation and RT-qPCR analysis. The remaining cultures were treated with NAG and monitored over the next 6 days for gametocyte production. The GCR was calculated by dividing the day 6 gametocytemia by the ring stage parasitemia at the time of RNA collection.

### Genetic diversity

Genomic DNA was extracted from two 3 mm punches of dried blood spot (D0) using QIAamp DNA Mini Kit (Qiagen), eluted in 120 μl of distilled sterile water and used to analyze *gdv1* and *msp2* genotypes. For *gdv1* a 613 bp section of *gdv1* (bp 207–819) containing the target SNP (NGS_SNP.Pf3D7_09_v3.1378602)^29^ was PCR amplified using primers, *gdv1*-outer Fw and *gdv1*-seq Rv (Supplementary Table 5). The PCR reaction mix contained 1 × AccuPrime ™ PCR Buffer II (15 mM MgCl2), 200 nM of each primer, and 0.4 unit of AccuPrime™ Taq DNA Polymerase (Thermo Fisher Scientific) in addition to 2 μl of genomic DNA (gDNA) template and consisted of initial denaturation at 94 °C for 4 min, followed by 40–50 cycles at 94 °C for 1 min; 38 °C for 50 sec, and 60 °C for 2 min; with final extension at 72 °C for 8 min. The PCR product was purified using a PCR clean-up kit (Macherey–Nagel) and sequenced using primers *gdv1*-seq Fw and *gdv1*-seq Rv (Supplementary Table 5). For *msp2 a* nested PCR was used to distinguish the two major allelic families (FC27 and 3D7). First, the central polymorphic region was PCR amplified using family specific primers, *msp2*.Outer.M2-OF and* msp2*.Outer.M2-OR^66^ (Supplementary Table 5) and reaction conditions described above for *gdv1*. Next, 0.5 μl of the outer PCR product was used as template in a PCR with primer *msp2* 3D7/FC27.S1fw and either *msp2* FC27.M5rev or *msp2* 3D7.N5rev. PCR products were separated using 2% ethidium bromide-stained agarose gels and visualized under UV illumination.

### Statistical analysis

STATA v14^36^ was used for the Pearson correlation and ANOVA analysis, as well as the independent regression analysis to evaluate the association of the clinical parameters and the ln transformed parasite data (D0 parasitemia, gametocytemia and gametocyte conversion rates on D4 and D8)^71^. To determine whether the parameters were normally distributed, histograms and normal quantile plots were used and those parameters that were not, D0 parasitemia, gametocytemia and GCR on D4 and D8, were ln transformed and retested. The metabolomics data for the high and low GCR cohorts were compared by unpaired, two-tailed Student’s t-tests and corrected for multiple comparison FDR < 0.05 (Microsoft Excel 2013)^72^. The LysoPC ROC curve was calculated using SPSS v24^73^. ANOVA analysis followed by a post test for multiple comparisons (Prism GraphPad v7.05)^71^ was used to compare the clinical parameters, including LysoPC levels in the high and low GCR samples in 2016 and 2017. Gene expression levels in the high and low GCR cohort samples were performed in duplicate and the 2^−ΔΔC^_T_ calculated using *18s rRNA* or *Sbp1* as the endogenous control and RNA levels in ring stage *Pfgdv1.gfp.dd*.T1 parasites grown in the absence of Shld1 as the reference. Differences between the 2^−ΔΔC^_T_ values for the high and low cohorts were analyzed by ANOVA using a Kruskal–Wallis test followed a Dunn’s multiple comparison test (Prism GraphPad v7.05). RT-qPCR analysis comparing gene expression in *Pfgdv1.gfp.dd* parasites with and without Shld1 as well as 3D7.G_def_ parasites were performed in triplicate in at least 2 separate experiments and analyzed by two way or one way ANOVA, respectively, with a Bonferroni post-test to correct for multiple comparisons (Prism GraphPad v7.05). Fisher’s exact test was used to evaluate the frequency of *gdv1* and *msp2* alleles in the 2016 and 2017 high and low GCR samples and the Kruskal–Wallis test followed a Dunn’s multiple comparison test was used to compare the GCR of the *gdv1* and *msp2* alleles (Prism GraphPad v7.05). The four samples, two from each cohort, with both *gdv1* alleles and the 11 samples, 5 from the high and 6 from the low GCR cohort, with both *msp2* alleles were included in the analysis. All statistical tests were two sided and *P* values > 0.05 were considered non-significant (ns), *P* values ≤ 0.05 = *, *P* values ≤ 0.01 = **, *P* values ≤ 0.001 = ***, *P* values ≤ 0.0001 = ****.

### Reporting summary

Further information on research design is available in the Nature Research Reporting Summary linked to this article.

## Supplementary information

Supplementary Information

Reporting Summary

## Data Availability

All data generated during this study are included in this published article and its supplementary information files. The raw data files are available on request. PlasmoDB.org accession numbers, *ap2-g*, PF3D7_1222600; *ap2-g3*, PF3D7_1317200; *arginyl-tRNA synthetase* PF3D7_1218600; *gdv1*, PF3D7_0935400; *Pfge3*, PF3D7_1477700; *gexp5*, PF3D7_0936600;* hp**1*, PF3D7_1220900; *kahrp*, PF3D7_0202000; *msrp1*, PF3D7_1335000; *Pfs16*, PF3D7_0406200; *Pfs25*, PF3D7_1031000; *Sbp1*, PF3D7_0501300; *18s rRNA*, PF3D7_0725600.
