# Publisher Correction: *Plasmodium falciparum* sexual differentiation in malaria patients is associated with host factors and GDV1-dependent genes

**DOI:** 10.1038/s41467-019-10805-w

**Published:** 2019-06-21

**Authors:** Miho Usui, Surendra K. Prajapati, Ruth Ayanful-Torgby, Festus K. Acquah, Elizabeth Cudjoe, Courage Kakaney, Jones A. Amponsah, Evans K. Obboh, Deepti K. Reddy, Michelle C. Barbeau, Lacy M. Simons, Beata Czesny, Sorana Raiciulescu, Cara Olsen, Benjamin K. Abuaku, Linda E. Amoah, Kim C. Williamson

**Affiliations:** 1 Uniformed Services University of the Health Sciences, Bethesda, MD 20814 USA; 2 Loyola University Chicago, Chicago, IL 60660 USA; 3 Noguchi Memorial Institute for Medical Research, University of Ghana, Accra, Ghana; 4 School of Medical Sciences, University of Cape Coast, Cape Coast, Ghana; 5 Present Address: Uniformed Services University of the Health Sciences, Department of Microbiology and Immunology, 4301 Jones Bridge Rd, Bethesda, MD 20814 USA

**Keywords:** Differentiation, Infectious-disease epidemiology, Infectious-disease diagnostics, Malaria

Correction to: *Nature Communications* 10.1038/s41467-019-10172-6, published online 13 May 2019.

The original version of this Article contained an error in Fig. 6, in which the right panel showing gametocytemia in the 6th cycle was flipped horizontally. This has been corrected in both the PDF and HTML versions of the Article.

