# Supplementary material for: Plasmodium falciparum sexual differentiation in malaria patients is associated with host factors and GDV1-dependent genes
Source: Nat Commun. 2019 May 13;10:2140. doi: 10.1038/s41467-019-10172-6 (PMC6514009; doi:10.1038/s41467-019-10172-6)
Supplement: Supplementary file 1 — Supplementary Information [file 41467_2019_10172_MOESM1_ESM.pdf]

Supplementary Table 1: Characteristics of the study population*

Group	All	2016	2017
**Sample Size**	261	129	132
**Age (yr)**	6.29±3.91	5.85±3.45	6.72±4.29
**Male (%)**	59	61	57
**D0 Parasites/µl**	73438±87794	97607±96916	49826±70603
**" (range)**	1093-757112	2050-757112	1093-434666
**D0 Parasitemia (%)**	4.18±4.33	4.81±4.89	3.56±3.61
**D0 gametocytes (n^)**	1	1	0
**D0 stage V/µl[@]**	15±102	12±100	18±105
**Hb (g/dl)**	12.08±1.86	11.46±1.86	12.68±1.65
**Temp (^{0}C)**	38.57±1.16	38.53±1.19	38.62±1.14
**≥37.5 (%)**	84	84	83
**WBC x10^3/µl**	10.99±5.16	12.73±5.60	9.30±4.06
**D4 G'cytemia**	0.105±0.261	0.134±0.303	0.076±0.211
**D8 G'cytemia**	0.113±0.242	0.131±0.279	0.097±0.203
**D4 GCR (%)**	3.98±10.25	4.56±12.02	3.42±8.16
**D8 GCR (%)**	3.58±8.92	3.76±10.58	3.42±7.11

*plus-minus values are means ±SD
^n=number of samples
[@]calculated from the Pfs25 RT-qPCR (Fig. S1)

Supplementary Table 2: Multiple linear regression analysis

D4

Source	SS	df	MS	Number of obs = 246		
Model	41.429	5	8.286	Prob > F = 0.2671		
Residual	1536.74	240	6.403	R-squared = 0.0263		
				Adj R-squared = 0.0060		
Total	1578.17	245	6.441	Root MSE = 2.5304		
lnD4CR	Coef.	Std. Err.	t	P>|t|	[95% Conf. Interval]	
Age	0.065	0.05	1.3	0.193	-0.033	0.164
Hb	-.121	0.097	-1.25	0.212	-0.311	0.07
WBC	0.018	0.035	0.52	0.601	-0.051	0.088
lnD0P	0.053	0.165	0.32	0.748	-0.272	0.379
Temp	-.264	0.145	-1.82	**0.069**	-0.549	0.021
_cons	10.111	5.614	1.8	0.073	-0.948	21.17

Source	SS	df	MS	Number of obs = 245		
Model	161.724	5	32.345	Prob > F = 0.0000		
Residual	1182.48	239	4.948	R-squared = 0.1203		
				Adj R-squared = 0.1019		
Total	1344.21	244	5.509	Root MSE = 2.2243		
lnD4GC	Coef.	Std. Err.	t	P>|t|	Conf. Interval]	
Age	0.053	0.044	1.21	0.227	-.034	0.141
Hb	-.106	0.086	-1.24	0.217	-.275	0.063
WBC	0.015	0.031	0.49	0.627	-0.046	0.076
lnD0P	0.746	0.145	5.13	**0**	0.459	1.032
Temp	-.260	0.127	-2.04	**0.042**	-0.510	-0.009
_cons	5.79	4.935	1.17	0.242	-3.931	15.511

D8

Source	SS	df	MS	Number of obs = 226		
Model	67.464	5	13.493	Prob > F = 0.0614		
Residual	1384.93	220	6.295	R-squared = 0.0465		
				Adj R-squared = 0.0248		
Total	1452.4	225	6.455	Root MSE = 2.509		
lnD8CR	Coef.	Std. Err.	t	P>|t|	[95% Conf. Interval]	
Age	0.065	0.052	1.24	0.215	-0.038	0.168
Hb	-.048	0.1	-0.49	0.628	-0.245	0.148
WBC	-.028	0.037	-0.76	0.449	-0.101	0.045
lnD0P	0.549	0.171	3.21	**0.002**	0.211	0.887
Temp	0.037	0.148	0.25	0.805	-0.255	0.328
_cons	-2.329	5.722	-0.41	0.684	-13.607	8.949

Source	SS	df	MS	Number of obs = 226		
Model	269.356	5	53.871	Prob > F = 0.0000		
Residual	1065.65	220	4.844	R-squared = 0.2018		
				Adj R-squared = 0.1836		
Total	1335.01	225	5.933	Root MSE = 2.2009		
lnD8GC	Coef.	Std. Err.	t	P>|t|	[95% Conf. Interval]	
Age	0.044	0.046	0.96	0.336	-0.046	0.135
Hb	-.053	0.088	-0.6	0.547	-0.226	0.12
WBC	-.024	0.033	-0.74	0.462	-0.089	0.04
lnD0P	1.084	0.15	7.22	**0**	0.788	1.381
Temp	-.004	0.13	-0.03	0.978	-0.259	0.252
_cons	-4.506	5.02	-0.9	0.37	-14.398	5.387

Multiple linear regression analysis of the clinical parameters indicate a significant association of Day 0 parasitemia with gametocyte conversion rate (CR) on D8 and gametocytemia (GC) on both days 4 and 8 using STATA version 14. Independently, none of the other clinical parameters, age, temperature (temp), white blood cell count (WBC) or hemoglobin concentration (Hb) were significantly associated with CR or GC.

Supplementary Table 3: Interaction model, multiple linear regression analysis

D4 gametocyte conversion rate

Source	SS	df	MS	
Model	67.789	3	22.596	Number of obs = 249
Residual	1524.564	245	6.223	Prob > F = 0.0136
				R-squared = 0.0426
				Adj R-squared = 0.0308
Total	1592.352	248	6.421	Root MSE = 2.4945

lnD4CR	Coef.	Std. Err.	t	P>|t|	[95% Conf.	Interval]
lnD0P	11.252	4.613	2.44	0.015	2.166	20.34
Temp	-.085	0.165	-0.52	0.606	-.411	0.24
c.lnD0P#c.Temp	-.291	0.119	-2.44	**0.016**	-.527	-.056
_cons	2.456	6.336	0.39	0.699	-10.025	14.94

D8 gametocyte conversion rate

Source	SS	df	MS	
Model	106.722	3	35.574	Number of obs = 229
Residual	1348.334	225	5.993	Prob > F = 0.0006
				R-squared = 0.0733
				Adj R-squared = 0.0610
Total	1455.056	228	6.382	Root MSE = 2.448

lnD8CR	Coef.	Std. Err.	t	P>|t|	[95% Conf.	Interval]
lnD0P	15.031	4.746	3.17	0.002	5.679	24.38
Temp	0.289	0.171	1.69	0.092	-.047	0.626
c.lnD0P#c.Temp	-0.378	0.123	-3.08	**0.002**	-.620	-.136
_cons	-12.393	6.561	-1.89	0.06	-25.322	0.536

Linear regression analysis demonstrates that for D4 and D8 gametocyte conversion (CR) there is a significant interaction effect between D0 parasitemia and temperature using a continuous temperature model. STATA version 14 was used for the analysis and the *p* value for the interaction is boxed (lnD4CR *p*=0.016 and lnD8CR *p*=0.002).

Supplementary Table 4: Characteristics of the High and Low GCR Cohorts #*^

Group	High GCR	Low GCR
**Sample Size (n)**	20	20
**Age (yr)**	6.24±2.87	4.95±3.28
**Male (%)**	50	61
**D0 Parasites/ul**	81050±73858	28690±24259
**“ (range)**	2602-242039	4563-101412
**D0 Parasitemia (%)**	4.40 ±5.35	2.35±2.29
**D0 gametocytes (n^)**	0	0
**D0 stage V/μl[@]**	7.6±19.0	2.8±5.8
**Hb (g/dl)**	11.8±1.21	11.97 1.58
**Temp (^{0}C)**	38.33 ± 1.18	38.80±1.40
**≥37.5 (%)**	75	85
**WBC x10^3/μl**	11.39±6.43	11.25±4.89
**D4 G'cytemia**	0.72±0.59	0.00± 0.00
**D8 G'cytemia**	0.62±0.49	0.00±0.00
**D4 GCR (%)**	27.13±20.52	0.01±0.06
**D8 GCR (%)**	23.89±21.80	0.03±0.08

Forty samples were selected, 20 from each year, with a D0P (> 0.35%) and either a high GCR (D4 GCR > 4.9 %, 10 from each year) or low to undetectable GCR (D4 GCR <0.3 %, 10 from each year). Samples with microscopically detectable gametocytes on D0 were not included.
*plus-minus values are means ± SD
^ number (n)
[@]calculated from the Pfs25 RT-qPCR (Fig. S1)

Supplementary Table 5: Oligonucleotide primers

Transformation	Primer Name	Sequence (5'-3')
Pfgdv1.gfp.dd Construction	Pfgdv1.gfp.dd-Fw	TAATCTCGAGGATTTCTTAATTATTCTTGATAAAATATGG
	Pfgdv1.gfp.dd-Rv	TAATCCTAGGTTTATATGTACATTTTTCTTTTATATAATAATGAAT
Integration analysis	SingleCross1-Fw	TGGAAAAAGCCTTAAAATTTGG
	SingleCross1-Rv	GTAGACCCCATTGTGAG
	SingleCross2-Fw	ATAATACCGCGCCACATAGC
	SingleCross2-Rv	TGAACATGCTATATTCTTATG
**qRT-PCR**	**Primer name**	**Sequence (5'-3')**
arginyl-tRNA synthetase	PF3D7_1218600-Fw	AGCTAAAGAGATGCATGTTGGTCATT
	PF3D7_1218600-Rv	GAGTACCCCAATCACCTACATGA
18S rRNA	18SrRNAUniv.-Fw	GCTGACTACGTCCCTGCCC
	18SrRNAUniv.-Rv	ACAATTCATCATCATATCTTTCAATCGGTA
sbp1	PF3D7_0501300-Fw	GGCATCTGCAACTACCGAAT
	PF3D7_0501300-Rv	GCTTGAAAAACCGTCATCGT
kahrp	PF3D7_0202000-Fw	CATGGTGCAGGCTATTCAG
	PF3D7_0202000-Rv	TTCACCGTCATTTCCTTCATGC
gdv1	PF3D7_0935400-Fw	TAGGCGTCGAAATAGTGCTAGTAGAAA
	PF3D7_0935400-Rv	GTCCTCACAACCAGCATCATTAGTA
hp1	PF3D7_1220900-Fw	CGAAAGCTAATGAGACAAATGGT
	PF3D7_1220900-Rv	CGTCGGGGTGCTAAGGAAC
var (subtel)	PF3D7_0426000-Fw	TGACGACTCCTCAGACGAAG
	PF3D7_0426000-Rv	CTCCACTGACGGATCTGTTG
var (central)	PF3D7_0412400-Fw	ACCGCCCCATCTAGTGATAG
	PF3D7_0412400-Rv	CACTTGGTGATGTGGTGTCA
ap2-g3	PF3D7_1317200-Fw	ATCTGGGAGGAGGTGGGTAG
	PF3D7_1317200-Rv	TTAGCTTGTGCCTGACCAAA
ap2-g	PF3D7_1222600-Fw	TGGTGGTAATAAGAACAACAGAGGT
	PF3D7_1222600-Rv	CCATCATAATCTTCTTCTTCGTCG
msrp1	PF3D7_1335000-Fw	TACCAGGTGCCTTATCAAGTG
	PF3D7_1335000-Rv	CTTGGTTGTGATTCGGTTGATG
gexp5	PF3D7_0936600-Fw	GTGGTTGTTTGAGAAGTGGTGA
	PF3D7_0936600-Rv	ACAGAATCCGTTTGAGATGATGA
Pfge3	PF3D7_1477700-Fw	ACGATACCCCACGAAATGAC
	PF3D7_1477700-Rv	CAACCATCCCATCCCTATTG
Pfs16	PF3D7_0406200-Fw	TTCTTCGCTTTTGCAAACCT
	PF3D7_0406200-Rv	AGCATGAAGAGAGGCACCTG
Pfs25	PF3D7_1031000-Fw	TCTTTTCCTTTTCATTCAACTTAGCA
	PF3D7_1031000-Rv	CCACTCATCTGAATTAAAAATCCTCTT
	PF3D7_1031000-1RvCL	TCCAAATGACCACTCATCTGAA
**allele analysis**	**Primer Name**	**Sequence (5'-3')**
gdv1	gdv1-outer Fw	TGGTTGTGAGGACTTGGTACA
	gdv1-seq Fw	GAAGAGATGGGAAACTTTTTACAAGA
	gdv1-seq Rv	GGCTTTTTCCATTTTCAAGTGT
msp2	msp2 Outer M2- OF	ATGAAGGTAATTAAAACATTGTCTATTATA
	msp2 Outer M2- OR	CTTTGTTACCATCGGTACATTCTT
	msp2 3D7/FC27 S1fw	GCTTATAATATGAGTATAAGGAGAA
	msp2 FC27 M5rev	GCATTGCCAGAACTTGAA
	msp2 3D7 N5rev	CTGAAGAGGTACTGGTAGA

Supplementary Figure 1

A.

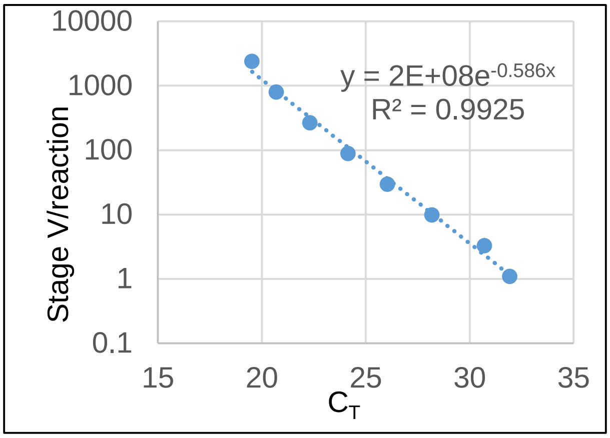

B.

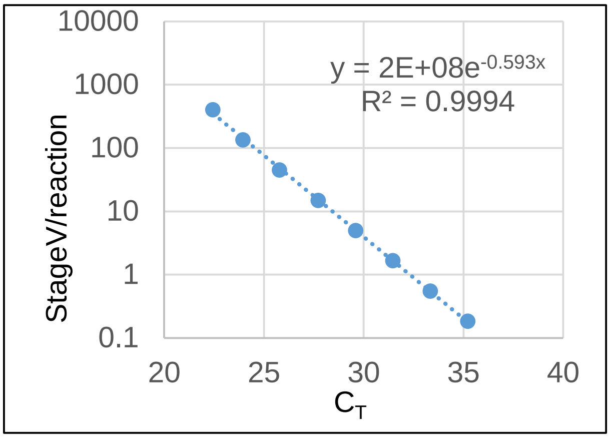

PAXgene			
C_T	C_T Mean	C_T SD	Stage V/Rx
19.55	19.53	0.032	2,402
20.71	20.70	0.018	801
22.23	22.31	0.112	267
24.23	24.15	0.105	89
26.09	26.04	0.061	30
28.20	28.18	0.029	10
30.61	30.71	0.141	3
31.95	31.93	0.019	1
36.96	36.69	0.390	NTC

NucleoZOL			
C_T	C_T Mean	C_T SD	Stage V/Rx
22.38	22.44	0.078	400.4
23.86	23.94	0.101	133.5
25.73	25.78	0.063	44.5
27.78	27.72	0.091	14.8
29.55	29.60	0.076	4.9
31.63	31.46	0.241	1.6
33.23	33.34	0.158	0.5
35.32	35.22	0.147	0.2
37.40	37.00	0.567	NTC

C.

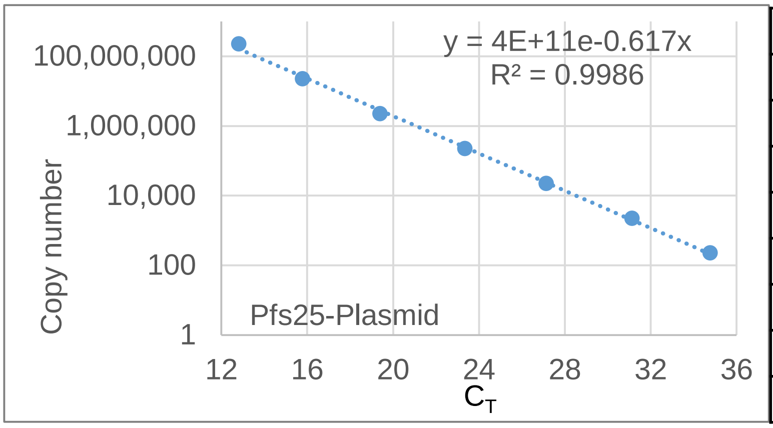

C_T Mean	C_T SD	Copies/Rx
12.81	0.193	454,000,000
15.77	0.128	45,400,000
19.37	0.074	4,540,000
23.33	0.199	454,000
27.13	0.025	45400
31.13	0.042	4,540
34.76	0.061	454
38.61	0.728	0

Supplementary Figure 1: Quantification of *Pfs25* transcript levels in stage V gametocytes. A-B) *Pfs25* RT-qPCR standard curves generated with RNA from uninfected human blood samples spiked with purified stage V gametocytes. RNA was isolated using either the PAXgene (A) or NucleoZOL (B) methods utilized for the 2016 or 2017 blood samples, respectively. C) *Pfs25* qPCR standard curve generated using plasmid containing a single copy of *Pfs25* (bp 24-128). The equations used to calculate stage V gametocyte numbers or transcript levels are shown on each graph. The associated tables list the mean and standard deviation of the *Pfs25* C_T values and the corresponding number of stage V gametocytes or *Pfs25* copies in each serial dilution as well as the non-template control (NTC).

Supplementary Figure 2

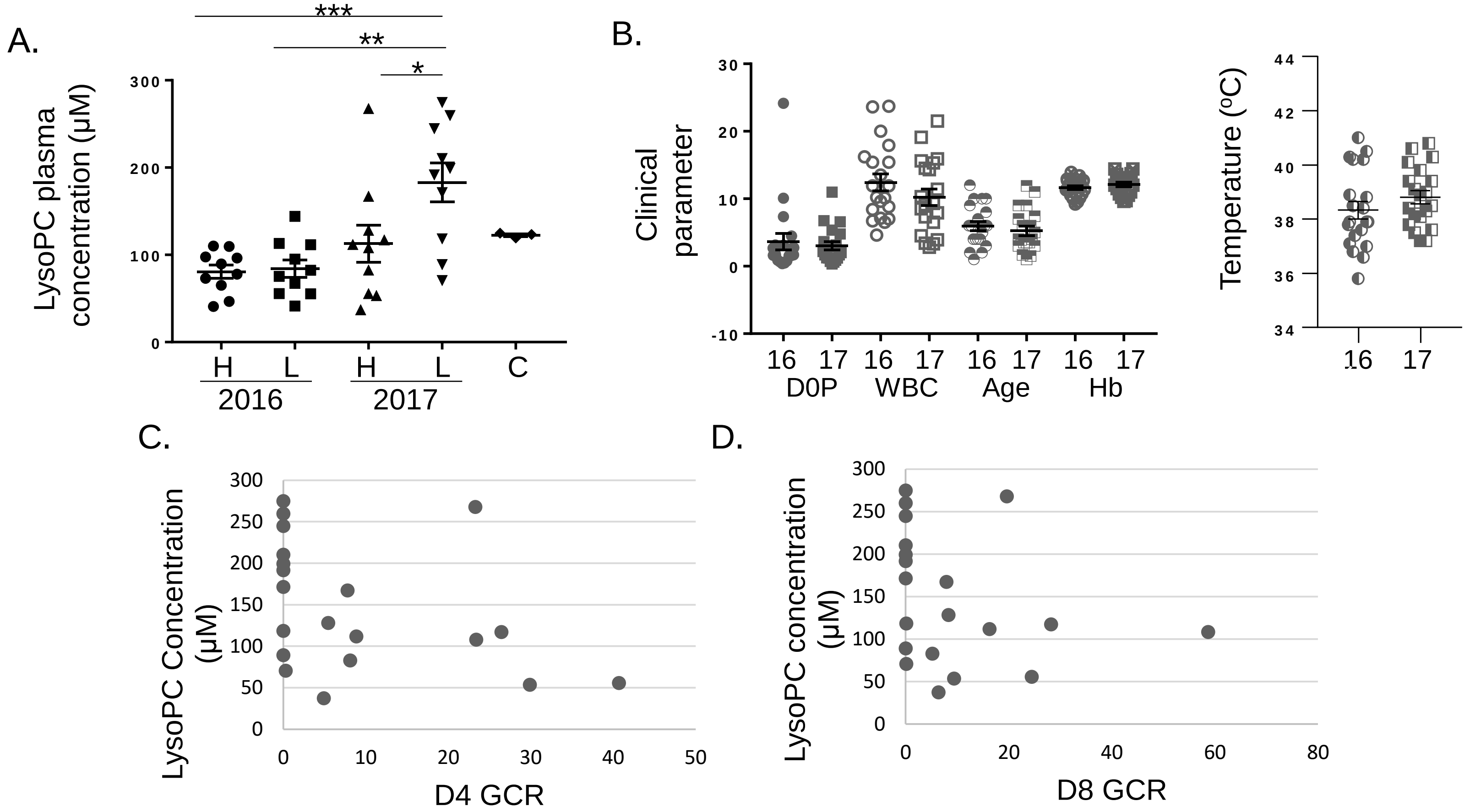

Supplementary Figure 2. Gametocyte conversion rate and LysoPC concentration. A) The total concentration of all 13 LysoPC isoforms tested are plotted for each of the samples in the 2016 and 2017 high (H) (n=20) and low (L) (n=20) GCR cohorts and control serum from North American volunteers (C) (n=3). Significance was determined using a one-way ANOVA and Tukey's test for multiple comparisons and probability is indicated, $p<0.05$ (*), $p<0.01$ (**), $p<0.001$ (***). B) Comparison of clinical parameters. The day 0 parasitemia (D0P, %), white blood cell count (WBC, $x10^3/\mu l$), age (yr), hemoglobin level (Hb, g/dl) and temperature (^oC) of each of the subjects enrolled in the High and Low cohorts in 2016 (n=20) and 2017 (n=20) are plotted in the first graph. Temperature is plotted in the second graph, since the y axis value is different. The mean and SEM are indicated. No significant difference between the 2016 and 2017 samples was found for any of the 5 parameters using a one-way ANOVA and Bonferroni's test for multiple comparisons. C-D) For each individual in the high (n=10) and low (n=10) GCR cohorts in the 2017 malaria season, the total day 0 plasma concentration of the thirteen LysoPC isoforms included in the Biocrates AbsoluteIDQ® p180 plate is plotted against the D4 (C) or D8 (D) GCR, which was calculated from the duplicate ex vivo samples as described in methods.

Supplementary Figure 3

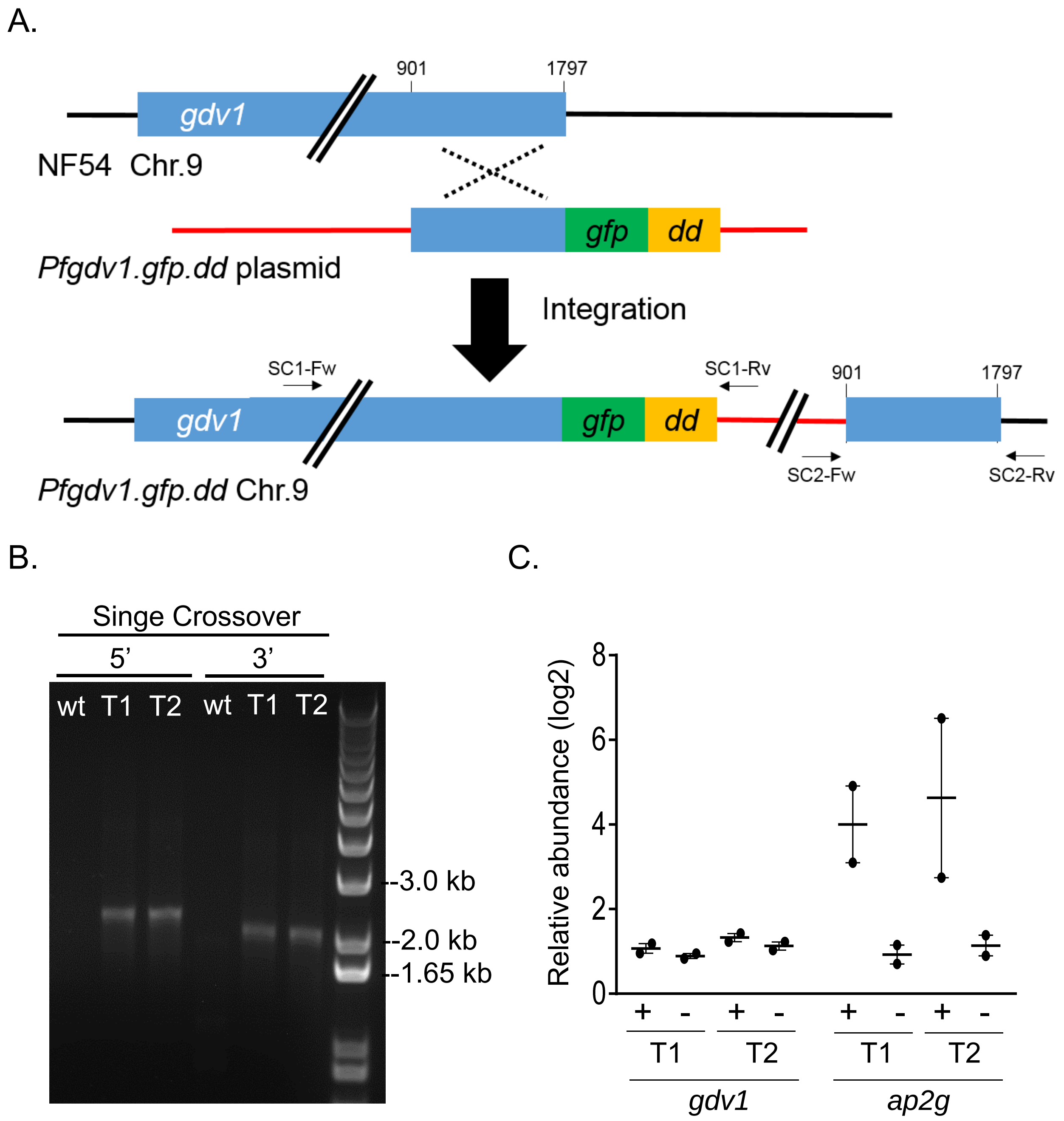

Supplementary Figure 3. PfGDV1 ligand dependent destabilization strategy and validation. A) Schematic of the wild type strain NF54 *gdv1* locus on chromosome 9 (NF54 Chr.9) before and after integration of the *Pfgdv1.gfp.dd* plasmid containing *gdv1* bp 901-1797 in frame with the coding region for green fluorescence protein (*gfp*) and the FKBP destabilization domain (*dd*). The primers used to evaluate chromosomal integration are indicated. B) Conformation of single crossover integration into the *gdv1* locus. Primers pairs (SC1-Fw and -Rv and SC2-Fw and –Rv, Supplemental Table 5) designed to amplify genomic DNA after *Pfgdv1.gfp.dd* plasmid integration were used to confirm single crossover integration from the 5' and 3' ends of the integration site, respectively, in both the T1 and T2 clonal lines. C) The relative abundance ($2^{-\Delta\Delta C_T}$) of *ap2-g* and *gdv1* transcripts were determined in RNA isolated from T1 or T2 schizonts cultured in the presence and absence of Shld1. The $2^{-\Delta\Delta C_T}$ was calculated using arginyl-tRNA synthetase (PF3D7_1218600) and the reference was the average ΔC_T for RNA from *Pfgdv1.gfp.dd*.T1& T2 schizont stage parasites grown in the absence of Shld1.

Supplementary Figure 4

A.

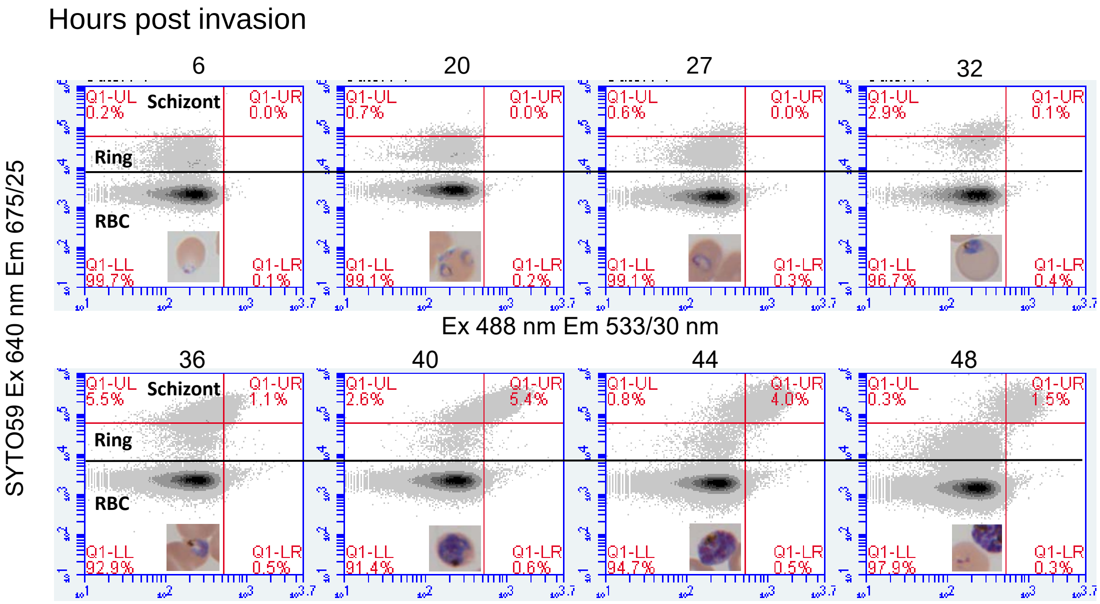

B.

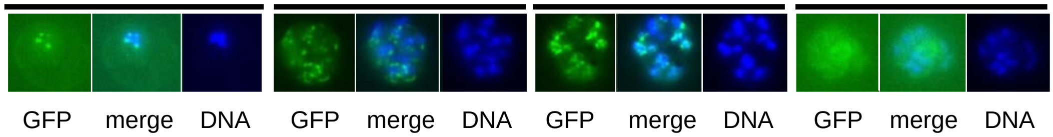

C.

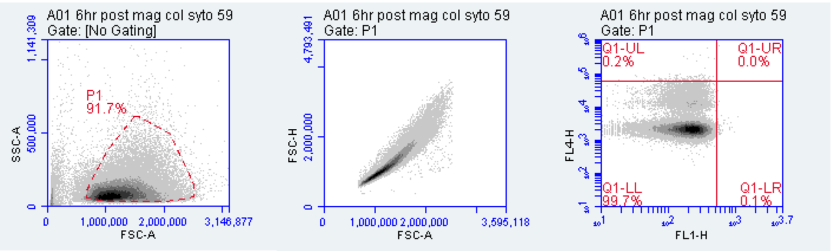

Supplementary Figure 4. GDV1 is expressed during schizogony. *Pfgdv1.gfp.dd*.T1 parasite development (A) and GDV1.GFP.DD expression (B) in the presence of Shld1 was monitored through the 48 h life cycle. At the indicated times post RBC invasion an aliquots of the culture were used to make a Giemsa-stained smear, stained with SYTO 59 or bisbenzimide for flow cytometry or directly observed by fluorescence microscopy. The increase in SYTO 59 signal (Ex 640 nm Em 675/25) monitors DNA replication as the parasite progresses through the cell cycle. C) Flow cytometry gating strategy to evaluate parasite stage. Synchronized, ring stage *Pfgdv1.gfp.dd* cultures (6±2 hpi) were incubated with SYTO 59 for 30 min and 100,000 events were evaluated by flow cytometry. Intact, infected and uninfected erythrocytes where selected based on the forward and side scatter profile (gate P1), then screened for monomers using the forward scatter height and area profile. Since there were no obvious multimers the fluorescence of all the cells in the P1 gate were used for analysis through the time course. The gate for DNA replication (schizont stages) was set just above the SYTO 59 signal (Ex 640nm/Em 675±25nm) for the 6±2 hpi rings and followed periodically for the next 48 hours.

Supplementary Figure 5

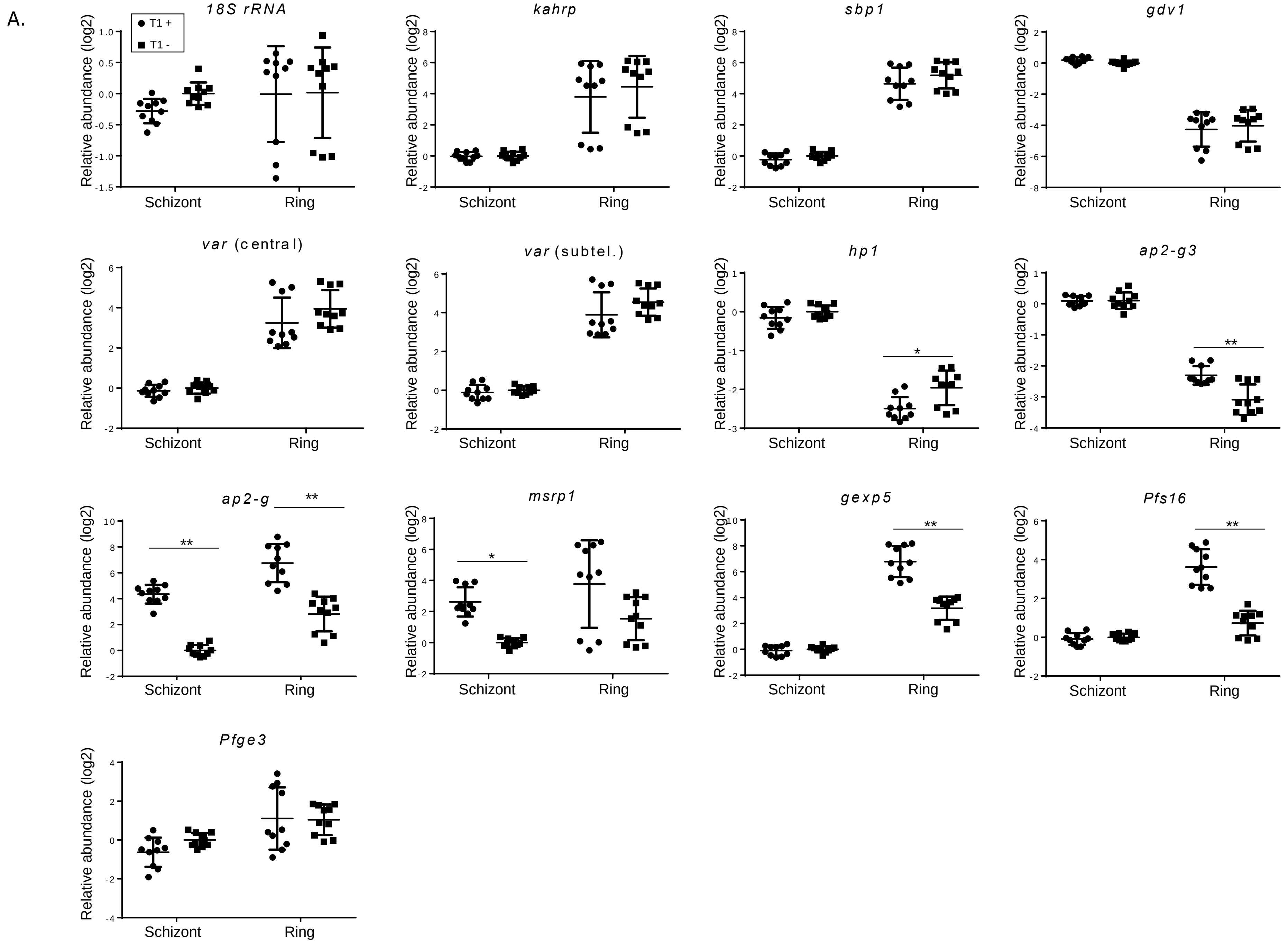

Supplementary Figure 5

B.

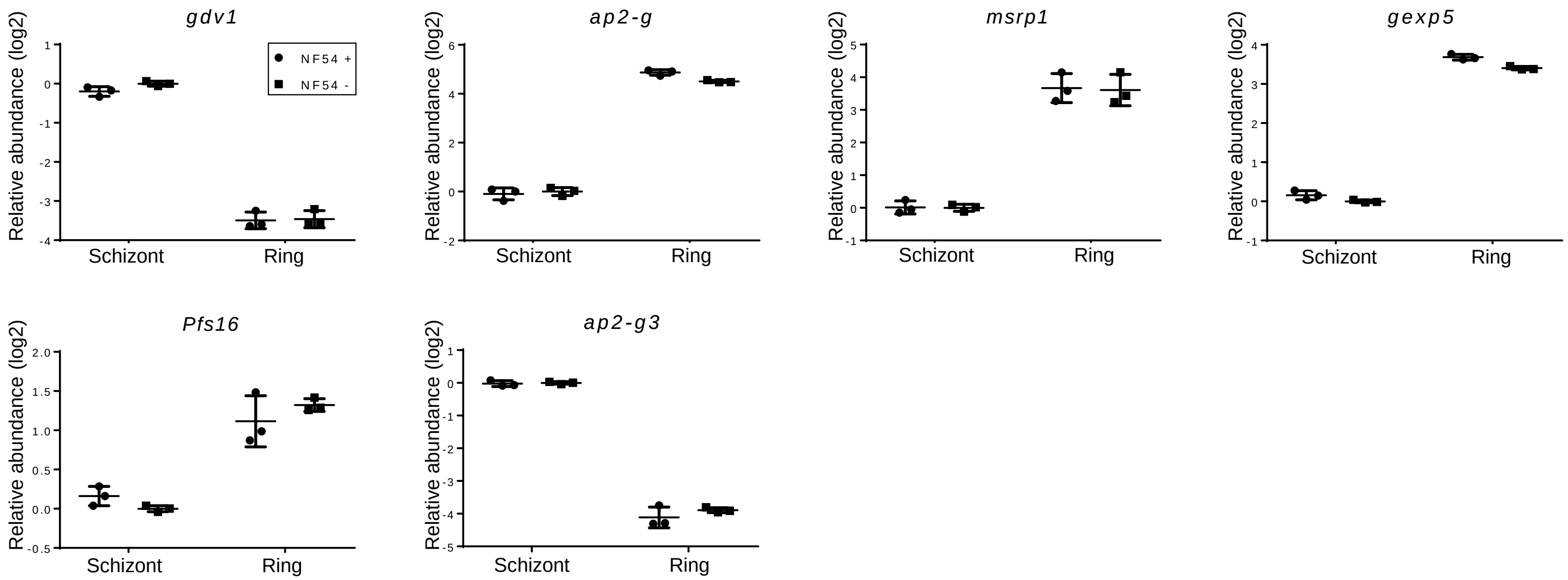

Supplementary Figure 5. GDV1-dependent increase in gametocyte-associated genes in *Pfgdv1.gfp.dd.*T1 parasites. The relative abundance of each gene ($2^{-\Delta\Delta C_T}$) in RNA from each individual flask of *Pfgdv1.gfp.dd*.T1 (A) or wild type NF54 (B) parasites at schizont or ring stages grown in the presence and absence of Shld1 are shown. The $2^{-\Delta\Delta C_T}$ was calculated separately for each of the 3 or 4 replicate flasks from 3 independent experiments (A) or triplicate flasks (B) using arginyl-tRNA synthetase (PF3D7_1218600) as the reference gene. The average ΔC_T of the indicated gene in RNA from all flasks of Shld1 minus *Pfgdv1.gfp.dd.*T1 schizont stage parasites from the same independent experiment was used as the baseline expression. A two-way ANOVA with Sidak's multiple comparisons test followed by a Bonferroni post-hoc test to correct for the analysis of 13 genes was used to determine significance and probability is indicated, $p \leq 0.05$ (*), $p \leq 0.01$ (**). The average fold change in the expression of each gene ($2^{-\Delta\Delta C_T}$) in *Pfgdv1.gfp.dd.*T1 or NF54 parasites is shown in Fig.7.

Supplementary Figure 6

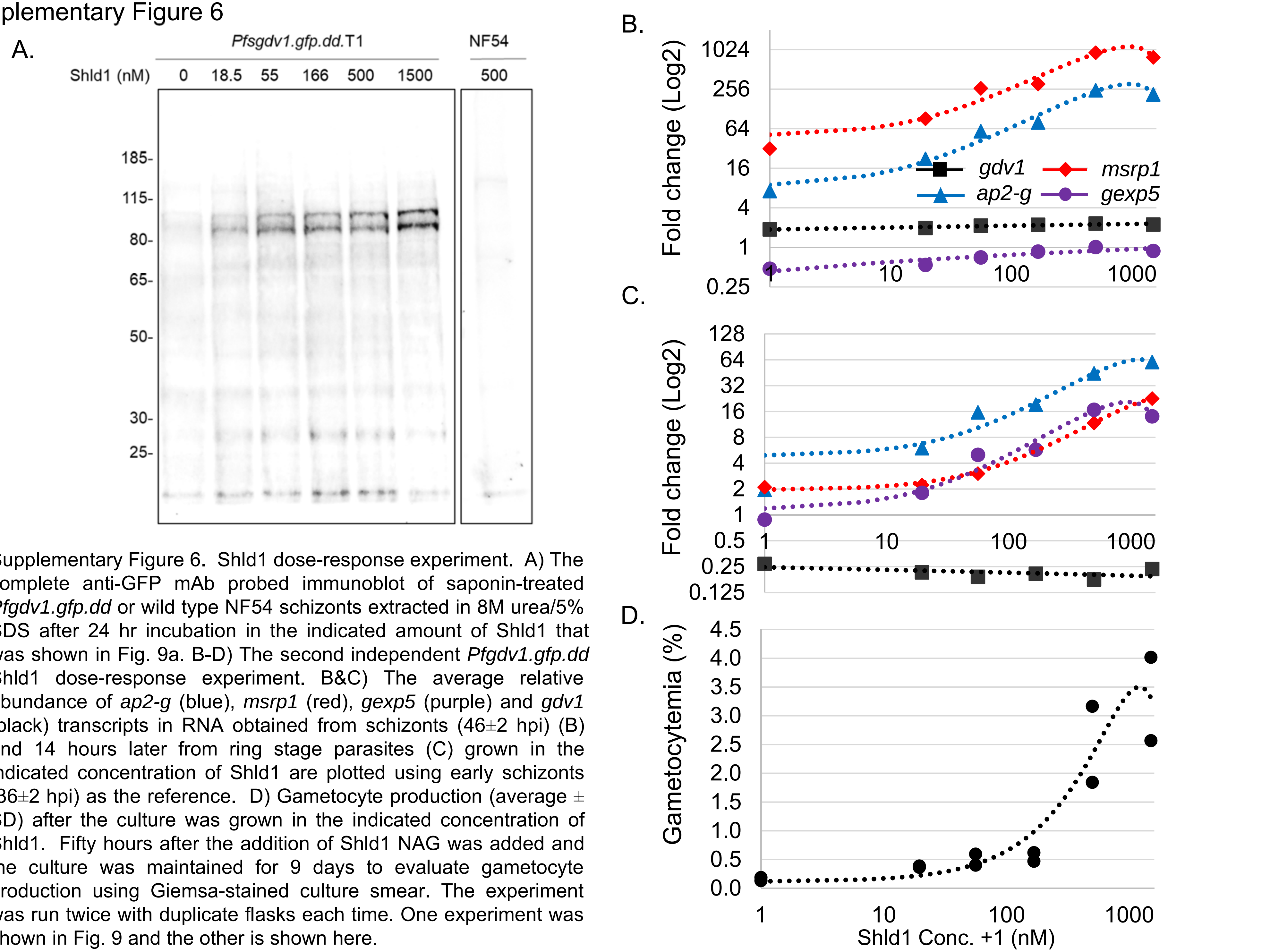

Supplementary Figure 6. Shld1 dose-response experiment. A) The complete anti-GFP mAb probed immunoblot of saponin-treated *Pfgdv1.gfp.dd* or wild type NF54 schizonts extracted in 8M urea/5% SDS after 24 hr incubation in the indicated amount of Shld1 that was shown in Fig. 9a. B-D) The second independent *Pfgdv1.gfp.dd* Shld1 dose-response experiment. B&C) The average relative abundance of *ap2-g* (blue), *msrp1* (red), *gexp5* (purple) and *gdv1* (black) transcripts in RNA obtained from schizonts (46±2 hpi) (B) and 14 hours later from ring stage parasites (C) grown in the indicated concentration of Shld1 are plotted using early schizonts (36±2 hpi) as the reference. D) Gametocyte production (average ± SD) after the culture was grown in the indicated concentration of Shld1. Fifty hours after the addition of Shld1 NAG was added and the culture was maintained for 9 days to evaluate gametocyte production using Giemsa-stained culture smear. The experiment was run twice with duplicate flasks each time. One experiment was shown in Fig. 9 and the other is shown here.
