# Supplementary material for: Plasmodium falciparum sexual differentiation in malaria patients is associated with host factors and GDV1-dependent genes
Source: Nat Commun. 2019 May 13;10:2140. doi: 10.1038/s41467-019-10172-6 (PMC6514009; doi:10.1038/s41467-019-10172-6)
Supplement: Supplementary file 2 — Reporting Summary [file 41467_2019_10172_MOESM2_ESM.pdf]

nature research

Corresponding author(s): Miho Usui

Reporting Summary

Nature Research wishes to improve the reproducibility of the work that we publish. This form provides structure for consistency and transparency in reporting. For further information on Nature Research policies, see Authors & Referees and the Editorial Policy Checklist.

Statistical parameters

When statistical analyses are reported, confirm that the following items are present in the relevant location (e.g. figure legend, table legend, main text, or Methods section).

n/a	Confirmed	
☐	☒	The exact sample size (*n*) for each experimental group/condition, given as a discrete number and unit of measurement
☐	☒	An indication of whether measurements were taken from distinct samples or whether the same sample was measured repeatedly
☐	☒	The statistical test(s) used AND whether they are one- or two-sided<br>*Only common tests should be described solely by name; describe more complex techniques in the Methods section.*
☐	☒	A description of all covariates tested
☐	☒	A description of any assumptions or corrections, such as tests of normality and adjustment for multiple comparisons
☐	☒	A full description of the statistics including central tendency (e.g. means) or other basic estimates (e.g. regression coefficient) AND variation (e.g. standard deviation) or associated estimates of uncertainty (e.g. confidence intervals)
☐	☒	For null hypothesis testing, the test statistic (e.g. *F*, *t*, *r*) with confidence intervals, effect sizes, degrees of freedom and *P* value noted<br>*Give P values as exact values whenever suitable.*
☒	☐	For Bayesian analysis, information on the choice of priors and Markov chain Monte Carlo settings
☒	☐	For hierarchical and complex designs, identification of the appropriate level for tests and full reporting of outcomes
☐	☒	Estimates of effect sizes (e.g. Cohen's *d*, Pearson's *r*), indicating how they were calculated
☐	☒	Clearly defined error bars<br>*State explicitly what error bars represent (e.g. SD, SE, CI)*

Our web collection on statistics for biologists may be useful.

Software and code

Policy information about availability of computer code

Data collection	Primer3 version 4.1.0, BD Accuri C6 Software, AxioVision v4.3.0 101, QCapture 2.9.13, QuantStudio Design&Analysis Desktop Software v1.3.1, ImageQuant LAS 4000 Version 1.2 Control Software
Data analysis	RPART package in R, SPSS version 24, STATA version 14, QuantStudio Design&Analysis Desktop Software, Microsoft Excel 2013, ImageJ 1.52a, Graphpad Prism 7

For manuscripts utilizing custom algorithms or software that are central to the research but not yet described in published literature, software must be made available to editors/reviewers upon request. We strongly encourage code deposition in a community repository (e.g. GitHub). See the Nature Research guidelines for submitting code & software for further information.

Data

Policy information about availability of data

All manuscripts must include a data availability statement. This statement should provide the following information, where applicable:

- Accession codes, unique identifiers, or web links for publicly available datasets
- A list of figures that have associated raw data
- A description of any restrictions on data availability

All data generated during this study are included in this published article and its supplementary information files. The raw data files are available on request.

PlasmoDB.org accession numbers, ap2-g, PF3D7_1222600; ap2-g3, PF3D7_1317200; arginyl-tRNA synthetase PF3D7_1218600; gdv1, PF3D7_0935400; ge3, PF3D7_1477700; gexp5, PF3D7_0936600; HP1, PF3D7_1220900; kahrp, PF3D7_0202000; msrp1, PF3D7_1335000; Pfs16, PF3D7_0406200; Pfs25, PF3D7_1031000; Sbp1, PF3D7_0501300; 18s rRNA, PF3D7_0725600.

Field-specific reporting

Please select the best fit for your research. If you are not sure, read the appropriate sections before making your selection.

☒ Life sciences ☐ Behavioural & social sciences ☐ Ecological, evolutionary & environmental sciences

For a reference copy of the document with all sections, see nature.com/authors/policies/ReportingSummary-flat.pdf

Life sciences study design

All studies must disclose on these points even when the disclosure is negative.

Sample size	The required sample size for the field work was estimated using the computer program (FPOWER), available in STATA software
Data exclusions	All samples were included in the assays except for the metabolomics and in vivo gene expression analysis. For these two experiments samples were categorized as High and Low gametocyte conversion rate (GCR) groups based on D0 parasitemia >0.35% and >4.9 % D4 GCR for High-GCR and a GCR <0.3 for the low GCR cohort. Samples were selected prior to the metabolomics and in vivo gene expression analysis.
Replication	All attempts at replication were successful.
Randomization	All samples were included in the assays, except for the metabolomics and gene expression analysis, which case samples were selected based on their D0 parasitemia and D4 GCR. They were not randomized
Blinding	High and Low GCR groups samples were blinded for metabolomics analysis.

Reporting for specific materials, systems and methods

Materials & experimental systems

n/a	Involved in the study	
☐	☒	Unique biological materials
☐	☒	Antibodies
☐	☒	Eukaryotic cell lines
☒	☐	Palaeontology
☒	☐	Animals and other organisms
☐	☒	Human research participants

Methods

n/a	Involved in the study	
☒	☐	ChIP-seq
☐	☒	Flow cytometry
☒	☐	MRI-based neuroimaging

Unique biological materials

Policy information about availability of materials

Obtaining unique materials	The remaining de-identified biological material from the field study will be available for assays that overlap with the scope of this study under a collaborative or material transfer agreement. The parasite lines used will be available upon request under a collaborative or material transfer agreement.

Antibodies

Antibodies used	Anti-GFP from mouse IgG1κ (clones 7.1 and 13.1)(Roche #11814460001, LOT:11063100),Anti-Histone H3 antibody from rabbit (abcam #ab1791, LOT:940500)
Validation	Anti-GFP from mouse IgG1κ validation information from Roche website:<br>Quality: Anti-GFP is tested for functionality and purity relative to a reference standard to confirm the quality of each new reagent Preparation<br>Purity: Both Anti-GFP mouse monoclonal antibodies (Clones 7.1 and 13.1) are >95% pure as determined by SDS-PAGE and ion-exchange HPLC analyses.

Anti-Histone H3 antibody validation information from abcam website:
Quality: Rabbit polyclonal to Histone H3 - Nuclear Loading Control and ChIP Grade

Eukaryotic cell lines

Policy information about cell lines

Cell line source(s)	The following reagent was obtained through BEI Resources, NIAID, NIH: Plasmodium falciparum, Strain NF54 (Patient Line E), MRA1000, contributed by Megan G. Dowler. Pfgdv1.gfp.dd and 3D7Gdef were produced in our laboratory.
Authentication	Genomic DNA from the parasite lines were analyzed for the presence or absence of the appropriate chromosomal insertions and GFP expression was evaluated by fluorescence microscopy. These parameters were reassessed periodically throughout the study.
Mycoplasma contamination	None of these line were tested for mycoplasma contamination.
Commonly misidentified lines (See ICLAC register)	None of these lines were used

Human research participants

Policy information about studies involving human research participants

Population characteristics	The following participant characteristics were assessed, age, gender, hemoglobin level, white blood cell count, and parasitemia. Microscopy was used to diagnose malaria. All study participants were given a standard curative dose of artemether-lumefantrine (20/120 mg/kg) or artesunate-amodiaquine (4/10 mg/kg) and scheduled for a follow up visit 7 days later.
Recruitment	Patients (age- <13 year) visiting the clinic were screened for malaria by microscopy. Following a positive diagnosis of uncomplicated malaria, the patient's parent or guardian was asked if they would be willing to participate in this study. If they agreed the study was explained in detail and they were asked to give informed consent.

Flow Cytometry

Plots

Confirm that:

☒ The axis labels state the marker and fluorochrome used (e.g. CD4-FITC).

☒ The axis scales are clearly visible. Include numbers along axes only for bottom left plot of group (a 'group' is an analysis of identical markers).

☒ All plots are contour plots with outliers or pseudocolor plots.

☒ A numerical value for number of cells or percentage (with statistics) is provided.

Methodology

Sample preparation	Cells were resuspended at 0.01% hematocrit (~1,000 cell/µl) in buffer A (154 mM NaCl, 9.27 mM glucose, 10 mM Tris-HCl, pH 7.4) with 500 nM SYTO 59 and incubated at RT in the dark for 20 to 30 min prior to analysis on an Accuri C6 flow cytometer (BD Biosciences, Franklin Lakes, NJ).
Instrument	Accuri C6
Software	BD Accuri C6 Software
Cell population abundance	>10,000 P. falciparum infected erythrocytes
Gating strategy	Intact parasite-infected and uninfected erythrocytes were selected using forward and side scatter and the cutoff for a single copy of genomic DNA was set from the maximum SYTO59 fluorescence of the synchronized ring-stage parasite population.

☒ Tick this box to confirm that a figure exemplifying the gating strategy is provided in the Supplementary Information.
